# A Diruthenium Metallodrug as a Potent Inhibitor of Amyloid-β Aggregation: Synergism of Mechanisms of Action

**DOI:** 10.1021/acs.inorgchem.3c03441

**Published:** 2023-12-20

**Authors:** Sara La Manna, Concetta Di Natale, Valeria Panzetta, Marilisa Leone, Flavia A. Mercurio, Irene Cipollone, Maria Monti, Paolo A. Netti, Giarita Ferraro, Aarón Terán, Ana E. Sánchez-Peláez, Santiago Herrero, Antonello Merlino, Daniela Marasco

**Affiliations:** † Department of Pharmacy, University of Naples “Federico II”, 80131 Naples, Italy; ‡ Department of Chemical, Materials, and Industrial Production Engineering (DICMaPI), University of Naples Federico II, 80125 Naples, Italy; § Interdisciplinary Research Centre on Biomaterials (CRIB), University of Naples Federico II, Istituto Italiano di Tecnologia, 80125 Naples, Italy; ∥ Institute of Biostructures and Bioimaging - CNR, 80145 Naples, Italy; ⊥ Department of Chemical Sciences, University of Naples “Federico II”, 80126 Naples, Italy; # CEINGE Biotecnologie Avanzate “Franco Salvatore” S.c.a r.l., 80131 Naples, Italy; 7 MatMoPol Research Group, Department of Inorganic Chemistry, Faculty of Chemical Science, Complutense University of Madrid, Avenida Complutense s/n, 28040 Madrid, Spain

## Abstract

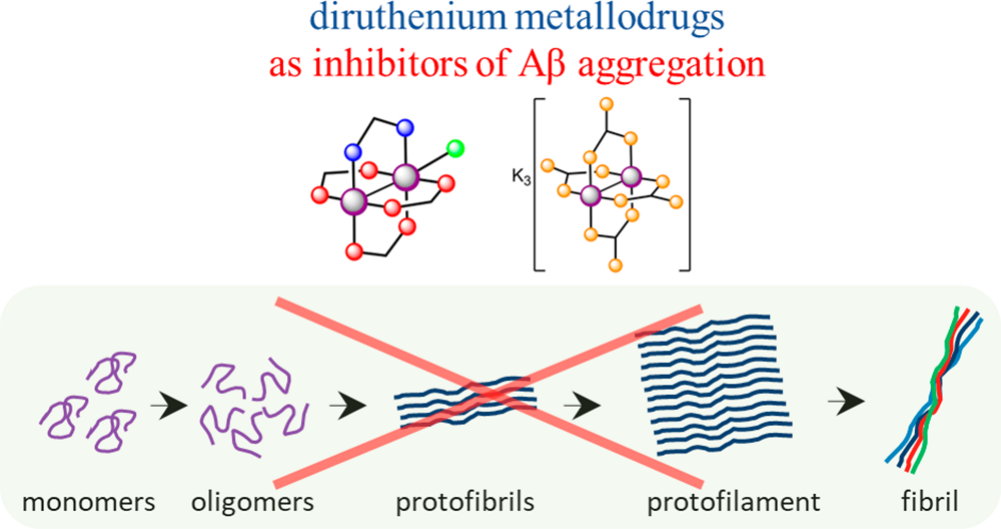

The physical and chemical properties of paddlewheel diruthenium compounds are highly dependent on the nature of the ligands surrounding the bimetallic core. Herein, we compare the ability of two diruthenium compounds, [Ru_2_Cl(D-*p*-FPhF)(O_2_CCH_3_)_3_]·H_2_O (**1**) (D-*p*-FPhF^–^ = *N,N′*-bis(4-fluorophenyl)formamidinate) and K_3_[Ru_2_(O_2_CO)_4_]·3H_2_O (**2**), to act as inhibitors of amyloid aggregation of the Aβ_1–42_ peptide and its peculiar fragments, Aβ_1–16_ and Aβ_21–40_. A wide range of biophysical techniques has been used to determine the inhibition capacity against aggregation and the possible mechanism of action of these compounds (Thioflavin T fluorescence and autofluorescence assays, UV–vis absorption spectroscopy, circular dichroism, nuclear magnetic resonance, mass spectrometry, and electron scanning microscopy). Data show that the most effective inhibitory effect is shown for compound **1**. This compound inhibits fiber formation and completely abolishes the cytotoxicity of Aβ_1–42_. The antiaggregatory capacity of this complex can be explained by a binding mechanism of the dimetallic units to the peptide chain along with π–π interactions between the formamidinate ligand and the aromatic side chains. The results suggest the potential use of paddlewheel diruthenium complexes as neurodrugs and confirm the importance of the steric and charge effects on the properties of diruthenium compounds.

## Abstract 2

Two bimetallic Ru-based compounds revealed their ability to act as inhibitors of amyloid aggregation of Aβ_1-42_ and its peculiar fragments, Aβ_1-16_ and Aβ_21-40_: they prevent fiber formation, and one of them completely abolishes the cytotoxicity of Aβ_1-42_, highlighting their potential application as antiamyloidogenic agents in Alzheimer’s disease.

## Introduction

Alzheimer’s disease (AD) is a chronic neurodegenerative disorder with a great social impact worldwide.^1^ Misfolding and extracellular aggregation of amyloid-β (Aβ) peptides and neurofibrillary tangles are recognized as the neuropathological hallmarks of AD.^2^

Aβ is predominantly an intrinsically disordered protein^3^ which self-assembles forming aggregates of different oligomerization levels and cytotoxic effects in AD.^4^ Monomeric Aβ exhibits unfolded random coil content with some α-helical and β-sheet stretches and nontoxicity.^5^ Aβ oligomers (AβOs) have been distinguished as types 1 and 2. Type 1 contains dodecamers or higher AβOs, that are reported to determine memory dysfunction, while type 2 is constituted by dimers or trimers, which are mainly involved in fibrillation and neuronal damage.^6,7^ Interestingly, a potential neuroprotective role of Aβ_1–42_ monomers was pointed out: they were able to defend primary cortical neurons against trophic deprivation and to protect mature neurons against excitotoxic death through the activation of the phosphatidyl-inositol-3-kinase (PI-3-K) pathway.^8−10^

One of the main areas of AD research has been focused on Aβ aggregation. This process occurs with Aβ isoforms of different lengths; the most abundant Aβ isoforms in the brain are Aβ_1–40_ and Aβ_1–42_ peptides. The main explored therapeutic approach is based on the inhibition of Aβ peptide aggregation,^11^ targeting the microscopic event of peptide oligomerization to prevent the macroscopic aggregation and associated toxicity.^12,13^ A crucial role in the Aβ peptide aggregation is played by the presence of a central hydrophobic core, constituted by residues Leu^17^-Ala^21^^14^ and a C-terminal domain containing several hydrophobic residues (Ala, Gly, Ile, Leu, and Val).^15−17^ Indeed, these regions are able to form internal β-sheet structures,^18^ while the N-terminal domain (residues 1–16) is not able to self-assemble under common conditions.^19^

Due to the complex nature of AD, multitarget drug design is emerging as a treatment strategy. Metallodrugs have been demonstrated to be a versatile opportunity due to the variety of the metal center oxidation states, coordination numbers and geometries, redox potentials of the species, and easy tuneability. Different complexes based on metals of *s*-, *p*-, *d*-, and *f*-block elements have been studied in AD as modulators of Aβ aggregation.^20−26^ Metal complexes through direct and indirect interactions with amyloid peptides can hamper or increase the formation of large-sized oligomers.^27^ Several studies have shown ruthenium-based complexes as a great alternative because they can inhibit aggregation and toxicity of Aβ.^28−31^ Although it depends on their particular composition, many Ru compounds present a strong affinity toward histidine side chains^31−33^ and thus can interact with Aβ at the N-terminal tail (that contains three His residues at positions 6, 13, and 14^34^) affecting the polypeptide’s conformation and ability to aggregate. Ru(II) complexes, like NAMI A, KP1019, and PMRU20, are able to bind the N-terminal domain^35^ or the entire Aβ_1–42_ peptide^36^ acting as potential aggregation inhibitors. The nature of the ligands can play an important role in the recognition process by Aβ_1–42_. Indeed, phenyl^37^ and tetraxylene bipyridine glycoluril ligands^38^ contribute to the inhibitory mechanism through π-stacking interactions. Similarly, heterodinuclear complexes containing both Pt(II) and Ru(II) metal ions bridged by 2,3-bis(2-pyridyl)benzoquinoxaline ligands exhibit a different mechanism of action where the inhibition of Aβ aggregation is due to Aβ_1–42_ conformational variations that force it to remain in the monomeric form.^39^ Very recently, it has been shown that NAMI-A has a dual inhibitory effect on both aggregation and membrane interaction of α-synuclein (α-syn) associated with Parkinson’s disease (PD).^40^ This complex abolishes the cytotoxicity of α-syn toward neuronal cells and mitigates neurodegeneration and motor impairments in a rat model of PD.

Within the group of Ru compounds, paddlewheel diruthenium complexes (Ru_2_^5+^) have emerged as a source of inspiration for drug design, because they offer a novel plethora of valuable molecular scaffolds that are not possible with mononuclear compounds. Most of the studied diruthenium compounds present the formula [Ru_2_Cl(O_2_CR)_4_] (R = aryl, allyl, or alkyl).^41^ These paramagnetic complexes are composed by two ruthenium atoms with a multiple metal–metal bond (bond order of 2.5) and two axial and eight equatorial positions that can be easily tuned.^42^

Diruthenium paddlewheel compounds have demonstrated their versatility in therapeutic areas, especially in cancer research.^43^ Although their mechanism of action remains unknown, their interactions with RNA^44^ or proteins show a strong structure–activity relationship.^45−47^ In general, diruthenium paddlewheel compounds show low stability or low solubility in aqueous medium.^48−53^ However, the introduction of one formamidinate ligand in the diruthenium coordination sphere permits obtaining stable and water-soluble derivatives.^46,53,54^ With these compounds, extensive structural studies to understand the molecular basis of Ru_2_ compounds in a living system have been carried out using the model protein hen egg white lysozyme (HEWL). The equatorial ligands modify the interactions with the side chains of the protein,^45^ the charge of the complexes permits modulation of the covalent and noncovalent bonding,^46^ and the medium can direct the axial or equatorial coordination of these species.^47^ Data indicate that the Ru–Ru bond remains stable upon the binding of the diruthenium species to the proteins, which make these compounds especially appealing (because of their particular distribution of coordination vacancies of the bimetallic core). However, this is not always the case for other binuclear paddlewheel compounds such as the dirhodium couterparts.^55−57^

**Scheme 1:**
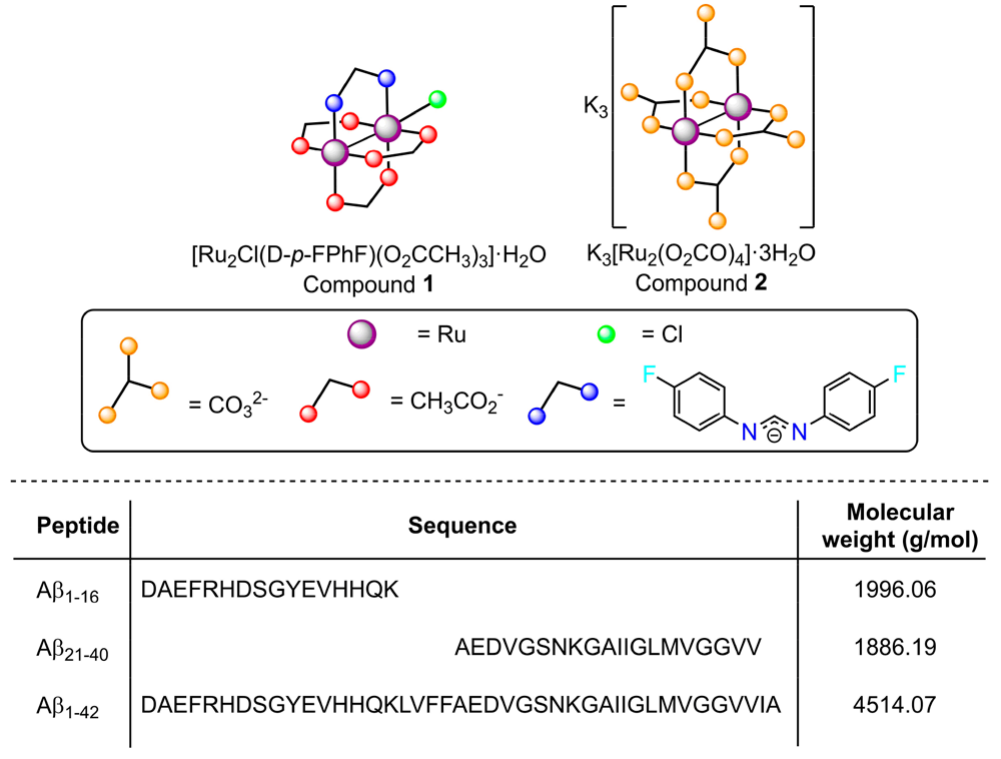
Structure of Diruthenium Compounds **1** and **2** and Aβ Peptides Sequences Used in This Work Water molecules have been omitted for clarity.

In order to explore the potential of these species, it was decided to test the compound [Ru_2_Cl(D-*p*-FPhF)(O_2_CCH_3_)_3_]·H_2_O (**1**) (D-*p*-FPhF^–^ = *N*,*N*′-bis(4-fluorophenyl)formamidinate) as an aggregation modulator of the Aβ_1–42_ peptide and its peculiar fragments Aβ_1–16_ and Aβ_21–40_. Also, the K_3_[Ru_2_(O_2_CO)_4_]·3H_2_O (**2**) complex was checked, bearing a different charge and steric hindrance with respect to compound **1** (Scheme 1), to compare the effect of these parameters on the aggregation modulator effect.

The different abilities of the two metal complexes to affect amyloid aggregation were analyzed by employing a wide range of spectroscopic and biophysical techniques. To the best of our knowledge, these data provide the first example of the use of paddlewheel diruthenium complexes as potent inhibitors of Aβ aggregation.

## Results and Discussion

### Effects of Ru_2_ Complexes on the Aggregation of Aβ Peptides

**Figure 1:**
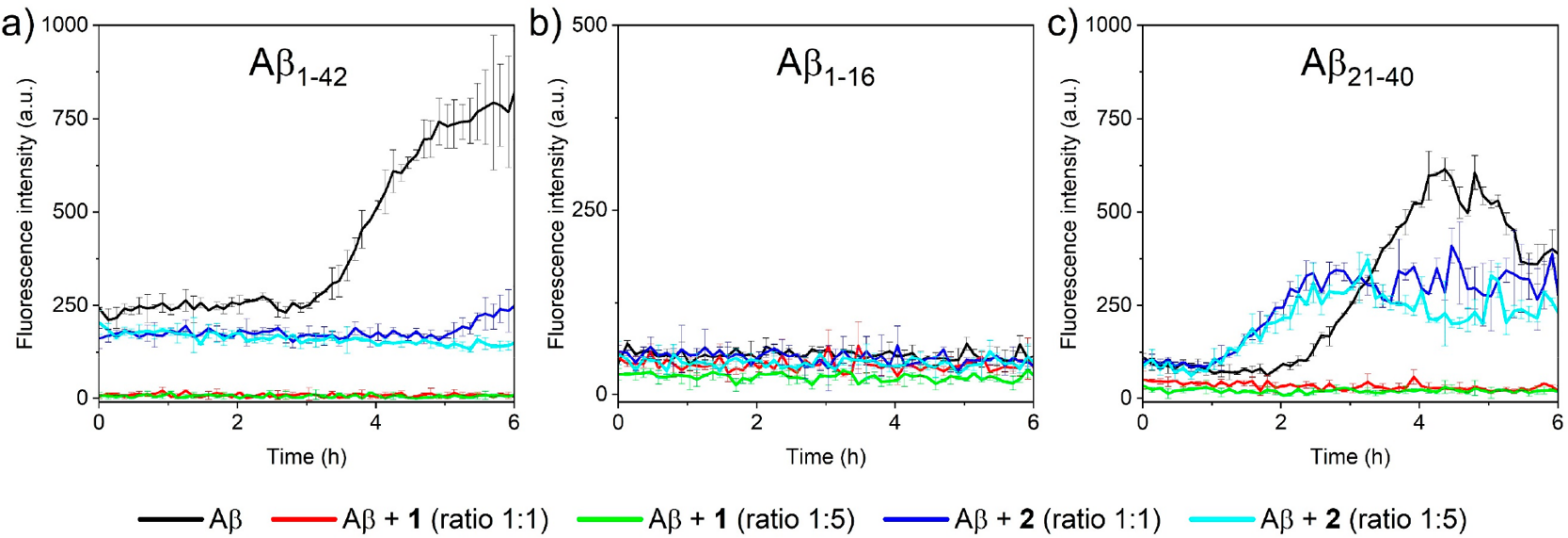
ThT fluorescence assay of peptides a) Aβ_1–42_, b) Aβ_1–16_, and c) Aβ_21–40_, in the absence and presence of compounds **1** and **2** at different Aβ:Ru_2_ molar ratios (1:1 and 1:5).

In order to examine the capacity of diruthenium compounds **1** and **2** to modulate the aggregation of Aβ peptides (Aβ_1–42_, Aβ_1–16_, and Aβ_21–40_), the thioflavin T (ThT) fluorescence assay was employed to monitor the amyloid aggregation^58^ in the absence and in the presence of compounds **1** and **2** (Figure 1).

Control experiments indicated insignificant fluorescence of compounds **1** and **2** in the ThT assay (Figure S1, ESI). Aβ_1–16_ peptide was not able to aggregate under the investigated experimental conditions since only a negligible fluorescence signal was observed for the Aβ_1–16_ peptide (Figure 1b) either in the absence or in the presence of compounds **1** and **2**.

The incubation of Aβ_1–42_ and Aβ_21–40_ peptides alone resulted in an increase of the fluorescence signal indicating fibril formation (Figures 1a and 1c). Equimolar and 5-fold molar excess of compounds **1** and **2** resulted in a drastic reduction of the fluorescence emission of Aβ_1–42_ and Aβ_21–40_ samples. For Aβ_1–42_, the presence of compound **1** completely suppresses the ThT signal, while compound **2** exhibits a less efficient reduction of fluorescence (Figure 1a). In the case of the Aβ_21–40_ peptide, compound **1** completely inhibits its aggregation (Figure 1c), while compound **2** reduces ∼50% of the ThT signal.

**Figure 2:**
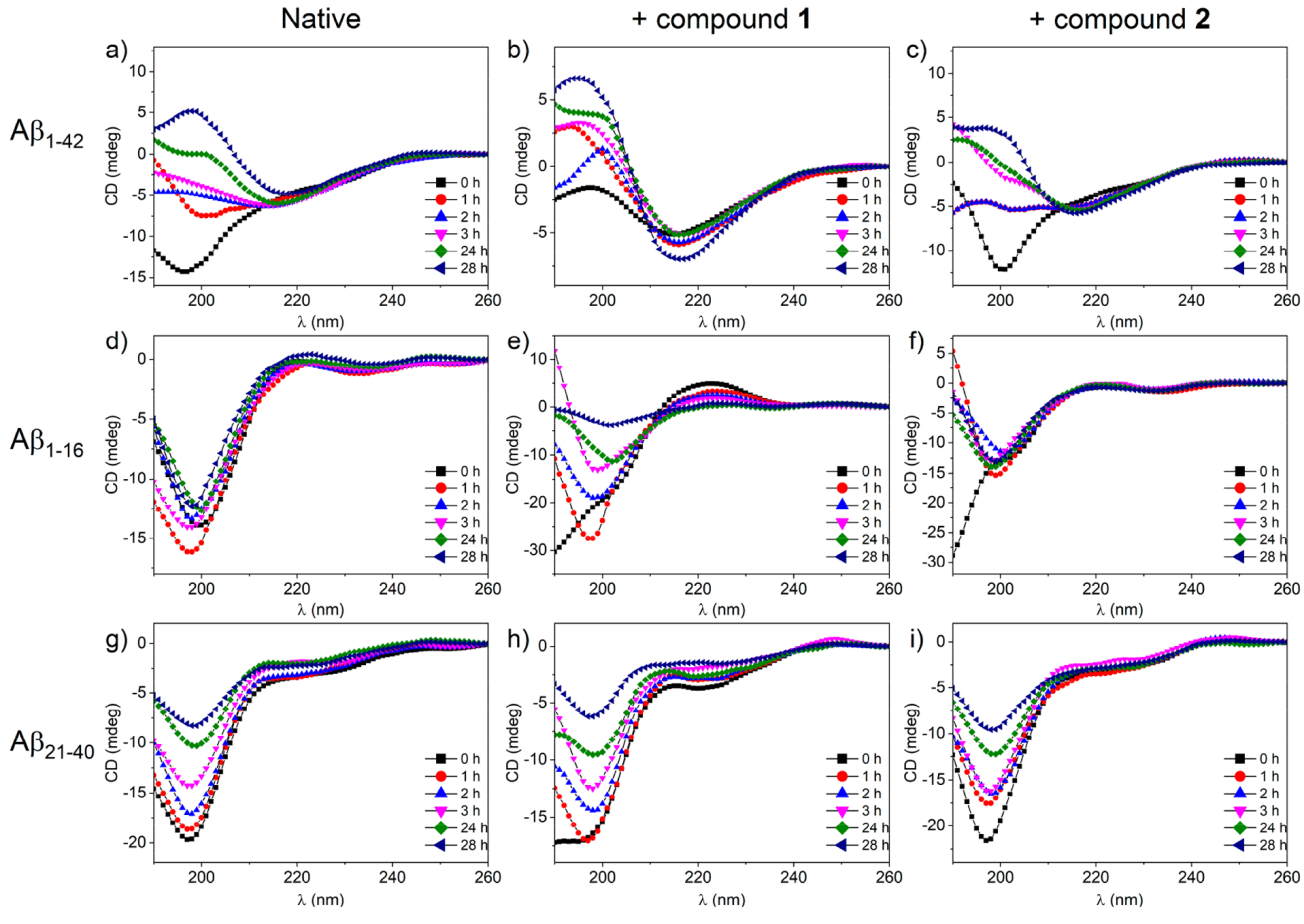
Conformational analysis over time of (a-c) Aβ_1–42_, (d-f) Aβ_1–16_, and (g-i) Aβ_21–40_ peptides in the absence and presence of compounds **1** and **2**. All samples were prepared at a 1:1 Aβ:Ru_2_ molar ratio.

Once the effect of compounds **1** and **2** on the aggregation was determined, in order to analyze the possible conformational changes of the Aβ peptides, circular dichroism (CD) experiments were carried out in the absence and presence of compounds **1** and **2** (Figure 2). Control experiments indicate that compounds **1** and **2** do not show CD signals (Figure S2, ESI).

The CD profile of the Aβ_1–42_ peptide (Figure 2a) shows a random coil conformation and exhibits a slow progressive transition toward a β-sheet structure over time, that is associated with a reduction of the Cotton effect.^59,60^ The presence of compound **1** determines a stabilization of the β-sheet structure already at *t* = 0 h (Figure 2b), and in line with ThT experiments, it seems to prevent further aggregation toward higher oligomers and fibers, as confirmed by the stability of the CD intensity over time. In contrast, compound **2** (Figure 2c) does not provide the same stabilization for the entire analysis time (28 h).

For Aβ_1–16_ and Aβ_21–40_ fragments (Figures 2d-2i), CD spectra reveal prevalent random content. The incubation of Aβ_1–16_ with compound **1** showed an aromatic positive band at the early stages of analysis, which suggests the formation of π–π stacking interactions (Figure 2e).

To obtain further insights into the interaction of compounds **1** and **2** with Aβ fragments, we carried out 1D ^1^H NMR and UV–vis spectroscopy studies. Aβ_1–42_ was not investigated by NMR because of the extreme sensitivity of its conformation with the extrinsic solution conditions, *i.e*. the presence of cosolvents, sample composition for multinuclear NMR, concentrations required for NMR assays and temperatures.^61^ On this basis, shorter protein fragments are often employed, in NMR investigations, as models to provide indirect proof of the mechanism of actions (MOAs) concerning the entire Aβ_1–42_ polypeptide.

**Figure 3:**
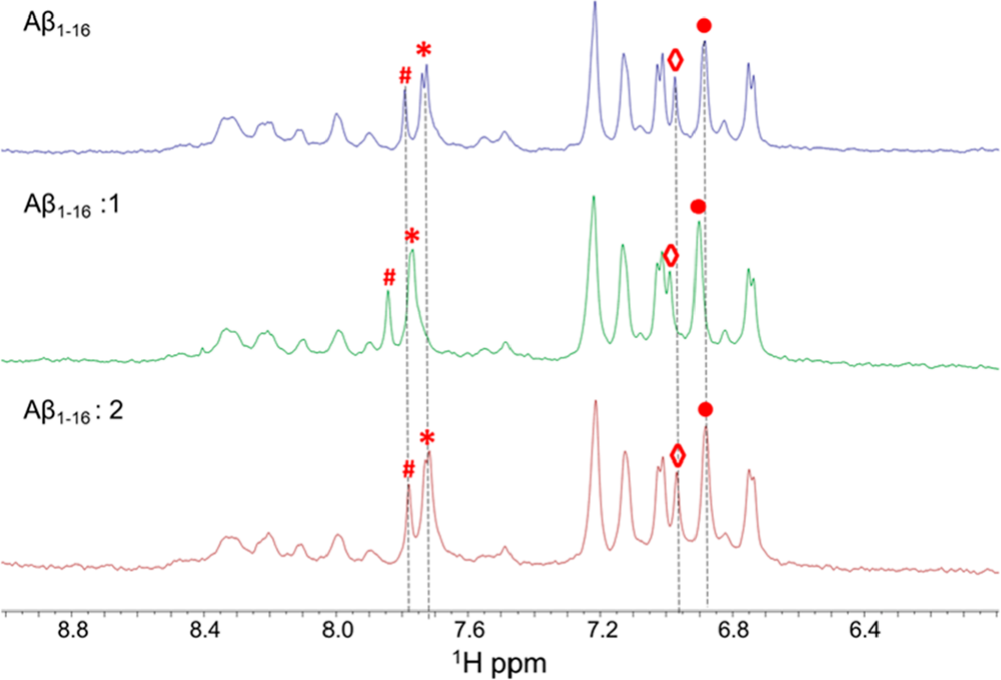
^1^H NMR spectra of Aβ_1–16_ in the absence (blue) and presence of compound **1** (green) or compound **2** (red). Peaks corresponding to the Aβ_1–16_ His residues (His^6^, His^13^, His^14^) are indicated by #, *, ◊, and ● symbols, whereas dashed lines highlight chemical shift changes.

Previous NMR studies pointed out the ability of other metal complexes to interact with Aβ peptides through His residues,^35,62^ and peaks from aromatic His protons can be easily identified in the spectrum by comparison with the literature.^62^ The Aβ_1–16_ spectra in the absence and in the presence of the paramagnetic compounds **1** and **2** (Figure 3) reveal a shifting of His signals^62^ (positions 6, 13, and 14) only in the presence of compound **1**. Other aromatic signals were not perturbed, suggesting a specific interaction between the His side chains and **1**. Conversely, the presence of compound **2**, which lacks aromatic ligands, does not provide relevant changes in the Aβ_1–16_ spectrum. The interaction between His side chains and diruthenium compounds is supported by crystallographic data available in our laboratories (unpublished data). Compound **2** produces anionic complexes in aqueous solution and is richer in electronic density than compound **1** that produces cationic species in solution.^46^ These differences could explain the differences in their reactivity with His residues.

Similar assays were carried out with the Aβ_21–40_ sequence, which lacks His residues. No relevant variations were observed either in the chemical shifts or in the intensities of the peaks (Figure S3). However, the spectrum contains signals largely deriving from monomeric and low molecular weight aggregates, as fast relaxing larger aggregates are not visible by the solution NMR technique. Thus, under the NMR experimental conditions, potential interactions of Aβ_21–40_ large aggregates with compounds **1** and **2** are likely not observable.^25,63,64^

The UV–vis spectra of the complexes **1** and **2** in the 240–700 nm region are characterized by the presence of bands due to ligand and metal-to-ligand charge transfer (LMCT) transitions.^45,46^ The absorbance variations observed upon increasing the amount of Aβ peptides led to guess a possible substitution of ligands around the bimetallic center (Figure S4, ESI) as it occurs when diruthenium species interact with the model protein HEWL.^45−47^ These variations are more evident for Aβ_1–16_ (Figures S4c and S4d, ESI), which contains three His residues and two aspartic and two glutamic acids, and less evident in the case of Aβ_21–40_, which only contains one aspartic and one glutamic acid, as potential ligands (Figures S4e and S4f, ESI). In this respect, it should be underlined that the obtained titration curves do not reach saturated values, suggesting variable stoichiometries of the interaction for both compounds.

To evaluate the formation of direct Aβ-Ru_2_ adducts, all three Aβ peptides were incubated with compounds **1** and **2** in a 1:5 molar ratio (Aβ:Ru_2_) and analyzed by electrospray ionization-mass spectrometry (ESI-MS) at 0 and 24 h of incubation. Spectra of isolated peptides and metal compounds were acquired as controls. As expected,^25^ under the employed experimental conditions, native Aβ peptides give rise to spontaneous fragmentation (b series elements). In the case of metal compounds, compound **1** loses the axial chloride ligand and one water molecule,^53^ while compound **2** loses the K^+^ counterions and three water molecules.

**Figure 4:**
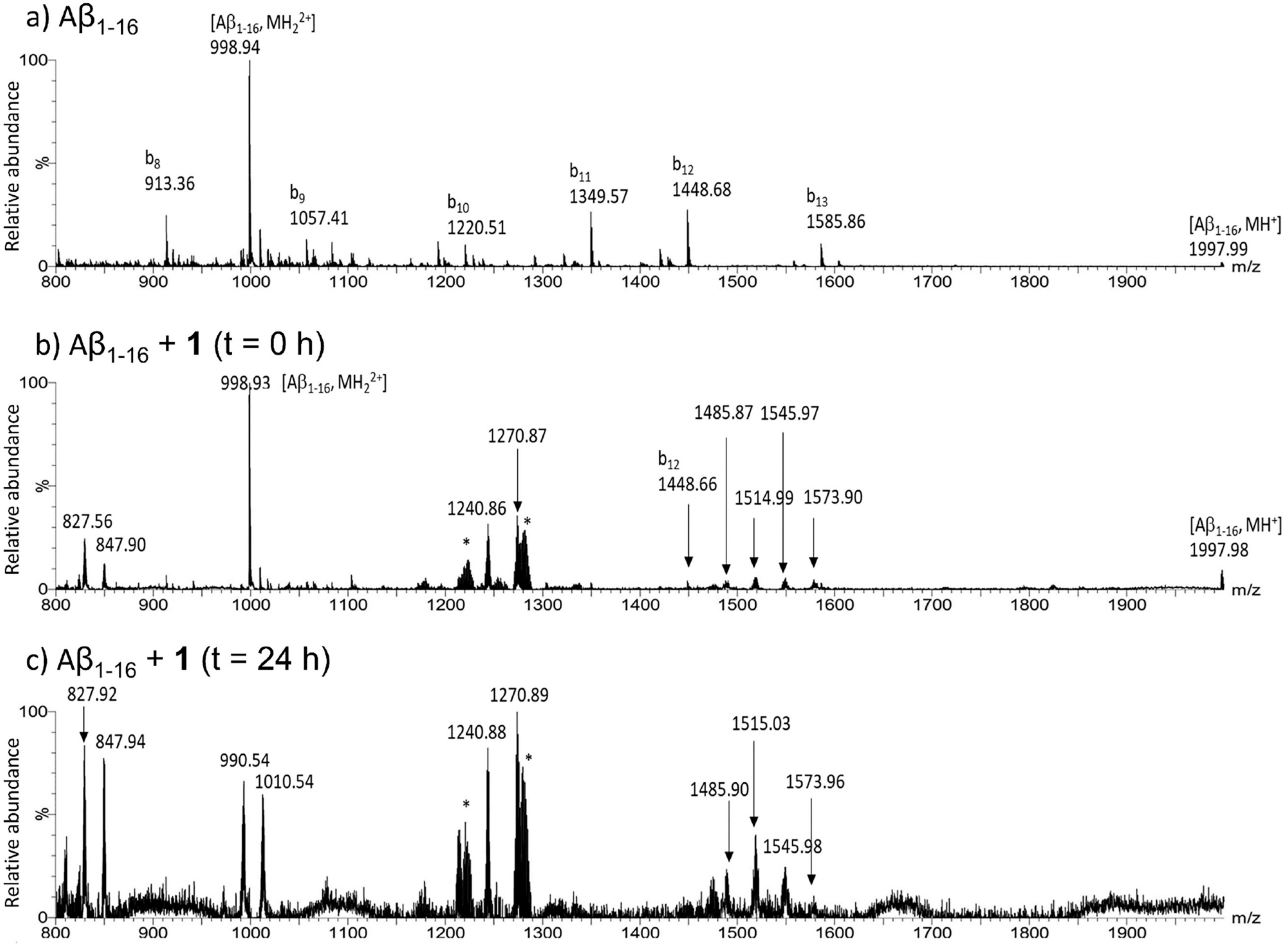
ESI-MS spectra of the Aβ_1–16_ peptide in the absence (a) and presence of compound **1** at *t* = 0 h (b) and upon 24 h of stirring (c). The peaks marked with bn derive from spontaneous source fragmentation of Aβ_1–16_ (b series elements). Asterisks (*) highlight the species present in the control (compound **1** alone).

After incubation with compound **1**, the spectral analysis of the Aβ_1–16_ peptide revealed that it can bind up to two fragments of compound **1**. In the case of the Aβ_1–16_-Ru_2_ 1:1 adduct, compound **1** loses, besides chloride and water molecules, one or two acetate ligands coordinated to the Ru_2_ core (Figures 4b and 4c and Table S1, ESI). The signals at *m*/*z* 827.56 and 1240.86 a.m.u. correspond to the triple and double charged ions generated by Aβ_1–16_ bound to one molecule of **1** losing two acetate ligands. Analogously, peaks at *m*/*z* 847.90 and 1270.87 a.m.u. derive from the triple and double charged adducts formed by the peptide and one molecule of **1** losing one acetate ligand (Table S1, ESI). Moreover, a cluster of signals at *m*/*z* 1485.87, 1514.99, 1545.97, and 1573.90 a.m.u. is assigned to double charged ions of Aβ_1–16_ bound to two molecules of **1** lacking four, three, two, or one acetate groups, respectively (Figure 4c and Table S1, ESI). It is noteworthy that the single (*m*/*z* 1997.98 a.m.u.) and the double (*m*/*z* 998.92 a.m.u.) charged signals of the unbound peptide disappear at the longest time (Figures 4a and 4c, and Table S1, ESI), suggesting that the peptide is completely involved in the binding of the diruthenium derivative. The presence of adducts endowed with different stoichiometries and high heterogeneities in ligand exchange is in line with results from NMR and UV–vis absorption spectroscopy and suggests that the metal compound can coordinate one or more side chains. In this respect, it could be possible to speculate that compound **1** could bind Asp or Glu side chains, in line with previous crystallographic data,^45^ and this equatorial ligand substitution can be accelerated by the previous axial coordination of the His side chains, as indicated by NMR data. This mechanism is supported by numerous studies of ligand substitution in diruthenium chemistry.^65^

Different results were found for Aβ_21–40_ and Aβ_1–42_ peptides (Figures S5 and S6 and Table S1, ESI). In the case of Aβ_21–40_, the signals *m*/*z* 944.03 and 1887.06 a.m.u., already present in the spectra of isolated Aβ_21–40_, persist in the presence of compound **1**, but two additional slight peaks at *m*/*z* 1246.94 and 1216.90 a.m.u., which occur only at *t* = 0 h, are observed. These peaks can be assigned to the adducts formed by Aβ_21–40_ and one molecule of **1** losing no acetate or one acetate ligand, respectively. These signals disappear after 24 h of incubation (Figure S5c, ESI), in agreement with the observed ThT profiles. However, the incubation of Aβ_1–16_ with compound **1** gives rise to an Aβ_1–16_-Ru_2_ adduct with the loss of one or two acetate groups. The different behavior exhibited by Aβ_1–16_ could also be explained with the presence of three His residues in the Aβ_1–16_ fragment and their axial coordination to promote the substitution of the acetate equatorial ligands by other carboxylate residues. For Aβ_1–42_, no meaningful signals of both isolated or metalated peptide were observed in the presence of compound **1** (Figure S6): these results, along with those derived from dynamic light scattering (DLS) analysis (see below), allow speculation that, in the presence of compound **1**, Aβ_1–42_ is not in its monomeric form (for the lack of signals of the peptide alone in Figure S6), but it likely forms soluble small aggregates, unable to ionize and to be detected in the employed mass conditions.

Similar experiments for compound **2** provided different results. The spectrum of the sample containing Aβ_1–16_ indicates that the peptide alone is the main species at both incubation times (*t* = 0 and 24 h), as demonstrated by the presence of the double charged ion at *m*/*z* 998.42 a.m.u. (Figure S7 and Table S2, ESI). In this case, the charge of complex **2** could be the reason why the interaction is not observed. However, at the longest time, 24 h, additional slight signals appeared at *m*/*z* 1094.79 and 1103.81 a.m.u. corresponding to double charged ions, which could be generated by a single peptide molecule carrying a Δm = +191.4 and +209.6 a.m.u., respectively, assignable to some portions derived from compound **2** that is losing anionic ligands with time^46^ thanks to the presence of His residues. In the same spectrum, fourthly (*m*/*z* 1046.85 and 1051.10 a.m.u.) and triply (*m*/*z* 1395.50 and 1401.20 a.m.u.) charged ions relative to the complexes showing 4183.4 and 4200.4 a.m.u. as molecular weights are also present. These data suggest that two peptide molecules could be bound to the same portion of the diruthenium compound. Conversely, in the case of Aβ_21–40_, beyond signals due to the peptide alone, no additional peaks are present even at 24 h. At this time, only an increase in adducts with potassium is evident (Figure S8 and Table S2, ESI). In this case, the charge of complex **2** and the lack of His residues can prevent a strong interaction between the diruthenium species and the peptide. Also, in the MS-spectrum of the sample of Aβ_1–42_ + compound **2**, there are only signals due to the presence of the peptide alone (Figure S9 and Table S1, ESI), which, different from compound **1**, persist at both times of incubation.

### Effects of Ru_2_ Complexes on the Autofluorescence and the Oligomeric States of Aβ Peptides

**Figure 5:**
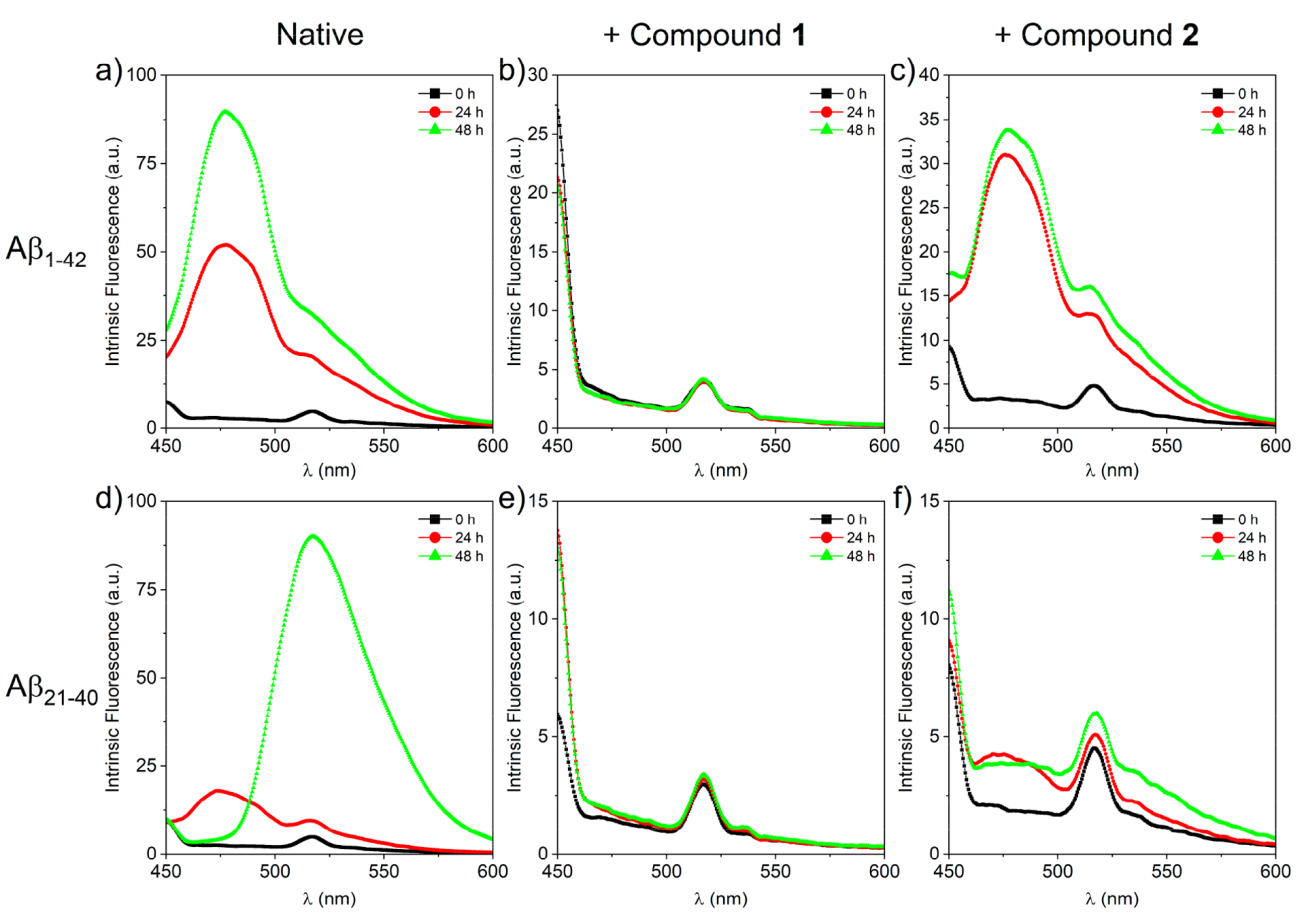
Fluorescence emission spectra of (a-c) Aβ_1–42_ and (d-f) Aβ_21–40_ peptides in the absence and presence of compounds **1** and **2** at different times (λ_ex_ = 440 nm and a 1:5 Aβ:Ru_2_ molar ratio).

The change of intrinsic fluorescence of Aβ peptides offers valuable information about the structure, folding, and dynamic of these molecules. The effect of complexes **1** and **2** on the autofluorescence of amyloid aggregates formation^66^ was evaluated by recording fluorescence emission spectra over time (Figure 5). The native Aβ_1–42_ exhibits a time dependent increase of emission intensities with maxima centered at two different wavelengths: 478 and 517 nm, respectively.^67^ Similar results are obtained for native Aβ_21–40_. In the presence of both compounds, a decreasing effect is observed for amyloids, with a greater effect for compound **1** (Figures 5b and 5e) when compared to compound **2** (Figures 5c and 5f). As expected, no autofluorescence was observed for Aβ_1–16_ alone or with complexes **1** and **2** (Figure S10).

**Figure 6:**
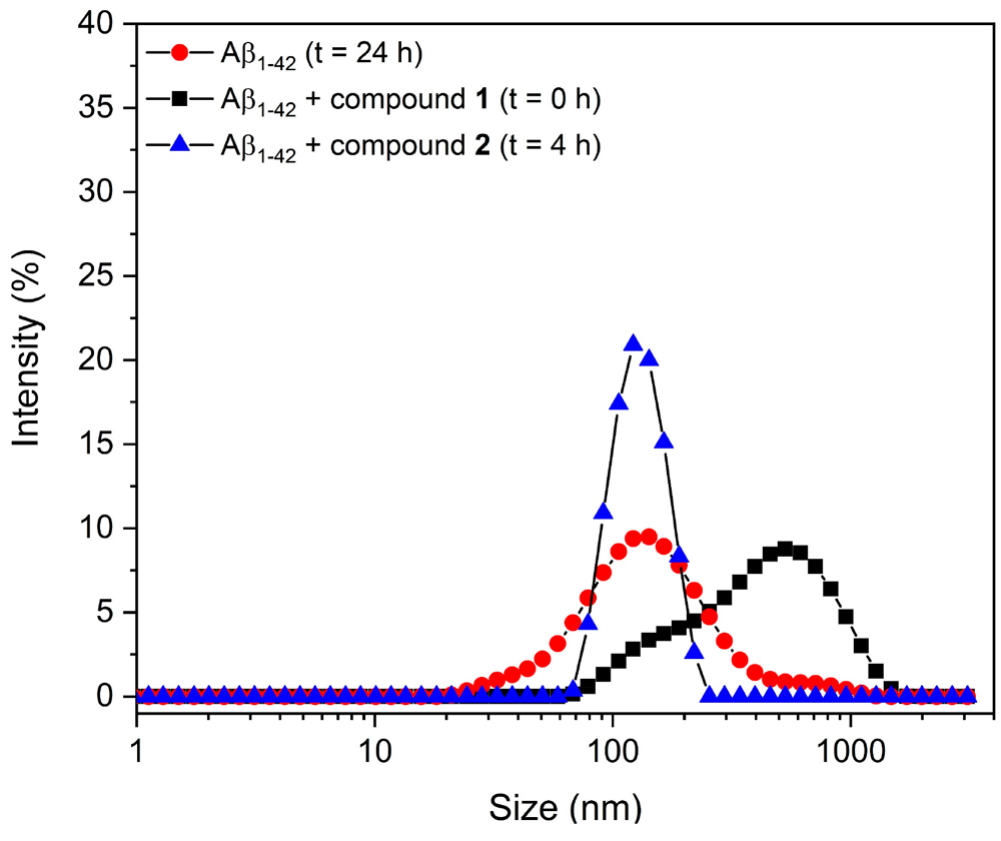
Overlay of size distribution by DLS of the Aβ_1–42_ peptide alone with compounds **1** and **2** at a 1:1 Aβ:Ru_2_ molar ratio, recorded at reported times of aggregation.

To further analyze the enhanced effect of diruthenium complexes **1** and **2** on Aβ_1–42_ aggregation, DLS was used to evaluate the Aβ aggregation dynamic process in a 24 h time-dependent inhibition study. The right autocorrelation for the Aβ_1–42_ fragment is achieved after 24 h^68,69^ with a peak centered at the diameter of 137 nm. Compound **1** anticipates the correct autocorrelation at *t* = 0 h, through the stabilization of an oligomeric form that exhibits a greater diameter, centered at 333 nm, which is maintained for 24 h (Figure 6). Observed DLS changes are consistent with the ability of compound **1** to stabilize Aβ_1–42_ oligomers, which are unable to further develop through fibrillation pathways, as reported for other inhibitory mechanisms.^69,70^ Conversely, compound **2**, which also anticipates the time of correlation at *t* = 4 h of incubation, provides a value for the diameter of 134 nm, which is close to that exhibited by Aβ_1–42_ alone before fibrillization. DLS analysis of Aβ_21–40_ is hampered by the small size of the peptide, which provides oligomers under the limit of detection (data not shown).

### Compound **1** Achieves Complete Rescue of Cell Proliferation in the Presence of Aβ_1–42_

**Figure 7:**
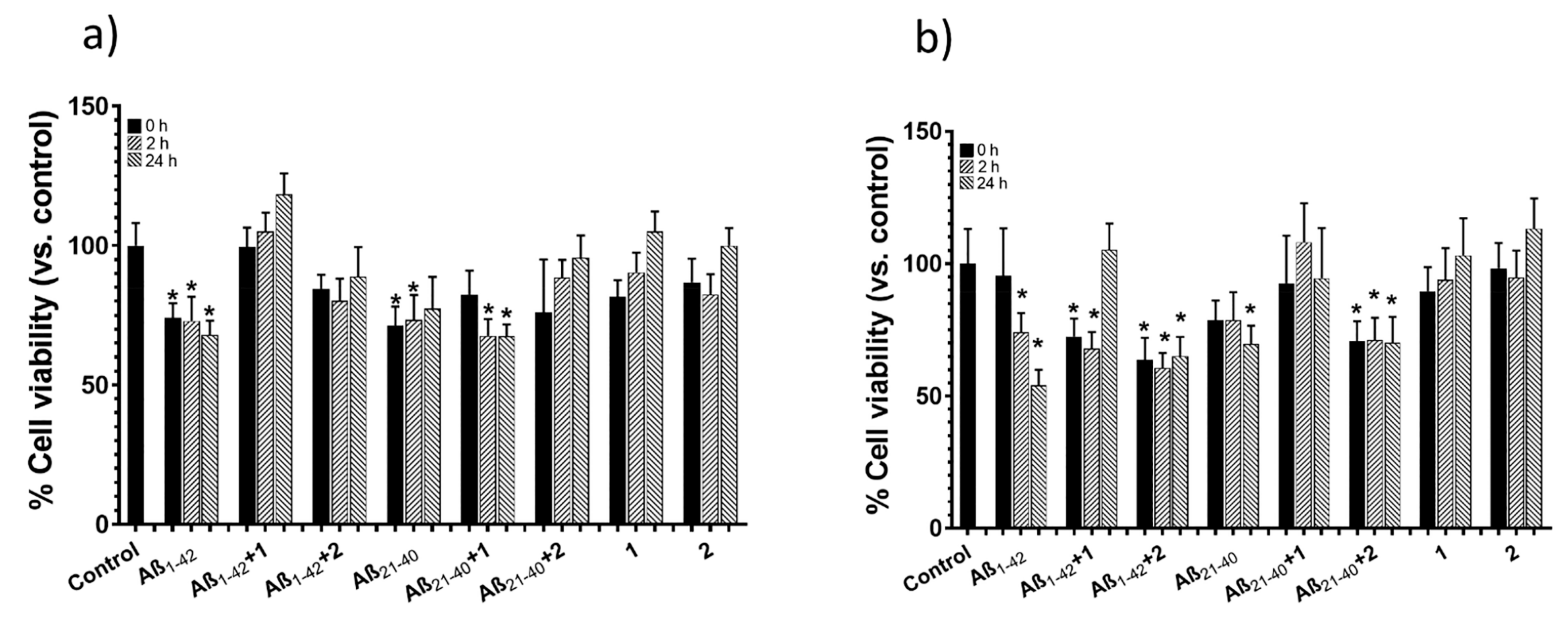
Effects of diruthenium complexes on cytotoxicity of Aβ peptides in a) SH-SY5Y and b) MCF7 cells: the MTT assay of Aβ_1–42_ and Aβ_21–40_ in the absence and in the presence of compounds **1** and **2** and diruthenium complexes alone, incubated under stirring, at *t* = 0, 2, and 24 h. Control refers to untreated cells as 100% of viable cells. Data are reported as mean ± standard deviation. Statistical comparisons were performed with a student’s unpaired t-test. *p < 0.05 with respect to control conditions.

To evaluate the effects of complexes **1** and **2** on the cytotoxicity of Aβ peptides, we performed a 3-(4,5-dimethylthiazol-2-yl)-2,5-diphenyltetrazolium bromide (MTT) assay at different times (0, 2, and 24 h) on SH-SY5Y cells (Figure 7a), human neuroblastoma cell lines commonly used as neuronal models. However, the cytotoxic mechanism of amyloids is common to a wide range of cells including breast cancer cells^71,72^ in which the evidence of accumulation of Aβ_1–42_ in tumors was reported in a mouse xenograft model of inflammatory breast cancer;^73^ thus, we performed the same assay on MCF-7 breast cancer cells (Figure 7b).

Aβ_1–42_ and Aβ_21–40_ alone exhibit different levels of cytotoxicity: indeed, Aβ_1–42_ at *t* = 0 shows a greater cytotoxic effect in neuronal cells even if, during time, in cancer cells it reached almost an ∼50% effect. On the other hand, Aβ_21–40_ presents similar toxicity in both lines with a minor effect with respect to Aβ_1–42_. In both assays, the presence of compound **1** with Aβ_1–42_ induces to a complete rescue of cell viability at *t* = 24 h, while for Aβ_21–40_, this effect is evident at all three times of analysis in MCF-7 (Figure 7b). An opposite slight effect is, instead, caused by compound **2** with Aβ_21–40_, which does not alter cell viability of MCF-7 cells (Figure 7b) while determining a partial enhancement of SHSY5 viability (Figure 7a). This slight difference could be due to the soft inhibitor effect of compound **1** on Aβ_21–40_ findable in ThT profiles (Figure 1c) which is evident in more sensitive neuronal cells.

As shown in Figure 7, both diruthenium complexes are not toxic at the concentrations used for these experiments, in line with our previous results with an analogous compound.^47^

### Compound **1** Drastically Modifies Amyloid Fiber Morphologies

**Figure 8:**
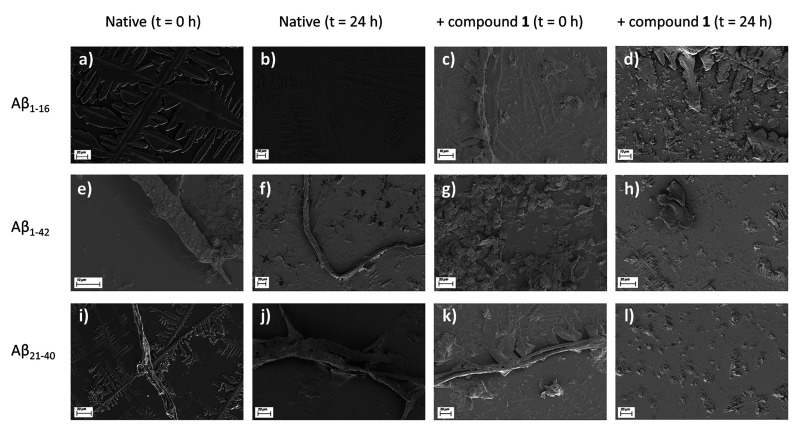
SEM microscopy images of (a-d) Aβ_1–16_, (e-h) Aβ_1–42_, and (i-l) Aβ_21–40_ peptides in the absence and presence of compound **1** at different times.

To assess the morphological changes of Aβ peptides in the presence of compound **1**, samples were visualized by using scanning electron microscopy (SEM). Unlike ThT or CD analysis, SEM microscopy cannot assess the monomeric state above all for peptides/proteins with a high propensity to aggregate, such as Aβ_1–42_ and Aβ_21–40_ sequences. Herein, we evaluated and compared the number and the maturity of the fibers in the presence and absence of metal complex **1**, at different times. In detail, the SEM analysis does not permit observation of fibers for the Aβ_1–16_ peptide (Figures 8a and 8d), while typical amyloid fibers are visible for Aβ_1–42_ at *t* = 0 h (Figure 8e and Table S3, ESI) as well as for Aβ_21–40_, especially for *t* = 24 h of aggregation (Figures 8i and 8j and Table S3, ESI). Compound **1** provides marked effects on the amyloid fibers: for Aβ_1–42_, a shortening and thinning effect is already observable at *t* = 0 h (Figure 8g and Table S3, ESI), and after 24 h, it determines the total absence of fibers (Figure 8h and Table S3, ESI). A less disaggregating effect is observed in the case of Aβ_21–40_: indeed well-structured fibers are preserved in the presence of compound **1** at *t* = 0 h, but they result completely dissolved after 24 h (Figures 8k and 8l and Table S3, ESI).

## Experimental Section

### Peptide and Metal Complex Syntheses

The Aβ_1–16_ peptide was synthesized as already reported,^24^ while Aβ_1–42_ and Aβ_21–40_ were purchased from NovoPro Bioscience, Inc. (Shanghai, China). All peptides were treated with 1,1,1,3,3,3-hexafluoro-2-propanol (HFIP) to guarantee a monomeric state, lyophilized, and stored at −20 °C until use.

For Ru complexes, all chemicals were of analytical reagent grade or of the highest purity commercially available. The precursor complex, [Ru_2_Cl(O_2_CCH_3_)_4_], was prepared and characterized in our laboratory, according to previously reported procedures,^74^ as well as the HD-*p*-FPhF ligand precursor.^75^ Complexes **1** and **2** were synthesized following the procedures reported in the literature.^53,76^

### Fluorescence Assays

ThT emission assays were carried out in black plates (96 well) under stirring on an Envision 2105 fluorescence reader (PerkinElmer). Measurements were collected every 7 min (λ_ex_ 440 nm and λ_em_ 483 nm). Assays were performed in duplicate at 25 °C employing a peptide concentration of 100 μM for Aβ_1–16_ and Aβ_21–40_ and 50 μM for Aβ_1–42_ in 50 mM NaCl and 20 mM phosphate buffer (pH 7.4), using a ThT final concentration of 50 μM for Aβ_1–16_ and Aβ_21–40_ and 5 μM for Aβ_1–42_. Different ratios with metal complexes (stock solutions 2 mM in water) were tested.

Autofluorescence assays of all peptides in the absence and in the presence of the metal compounds were carried out at the same concentrations used for the ThT assays, in a quartz cuvette with an optical path length of 10 × 2 mm, under stirring on a Jasco FP 8300 spectrofluorometer (λ_ex_ = 440 nm). Measurements were collected after 0, 24, and 48 h of stirring.

### Circular Dichroism (CD) Spectroscopy

CD spectra of Aβ_1–42_ at 50 μM and Aβ_1–16_ and Aβ_21–40_ at 100 μM, in 10 mM phosphate buffer pH 7.4, in the absence and presence of the metal compounds in a 1:1 peptide to complex molar ratio, were registered on a Jasco J-815 spectropolarimeter (JASCO, Tokyo, Japan), at 25 °C using a 0.1 cm path-length quartz cuvette. CD spectra were recorded over time using solutions prepared by the dilution of freshly prepared stock solutions of Aβ peptides (∼1 mM).^77^

### UV–Vis Spectroscopy

UV–vis titrations of compounds **1** and **2** with Aβ_1–42_, Aβ_1–16_, and Aβ_21–40_ were carried out employing BioDrop Duo UV Visible Spectrophotometers (Cambridge, United Kingdom). To a fixed concentration of metal compounds (50 μM), increasing amounts of peptides (2.5 μL of peptide stock solutions 1 mM in water, kept at 0 °C) were added. Reached ratios are reported in Figure S4. Each spectrum was registered (240 to 700 nm) upon the addition of the peptide.

### NMR Spectroscopy

NMR spectra were recorded on a Bruker Avance Neo 500 MHz instrument equipped with a cryoprobe at a temperature of 25 °C. NMR experiments were carried out with Aβ_1–16_ and Aβ_21–40_ in the absence and in the presence of compound **1** or compound **2** (1:1 peptide to metal compound molar ratio). In all samples, peptides and compounds were 250 μM in 550 μL of 10 mM sodium phosphate buffer pH 7.4 and 50 μL of D_2_O (deuterium oxide, 98% D, Sigma-Aldrich, Milan, Italy). 1D ^1^H NMR spectra were recorded with 16–128 scans; water suppression was achieved through excitation sculpting.^78^ The residual water peak at 4.75 ppm was used for chemical shift referencing. Spectra processing and analysis were conducted with TopSpin4.2.0 (Bruker, Italy).

### ESI-MS Analysis

Aβ-peptides were incubated with compounds **1** and **2** in 15 mM ammonium acetate (AMAC) buffer pH = 7.0, in a 1:5 ratio, and analyzed by native ESI-MS at 0 and 24 h of incubation. Mass spectra were acquired on a Q-ToF Premier (Waters, Milliford, MA, USA) mass spectrometer by direct injection at 10 μL/min, setting the source parameters at 3 kV for the capillary voltage and 42 kV for the cone voltage. The acquisition range was scanned from 100 to 5000 *m*/*z* in 1 s, and the raw data were processed with MassLynx 4.1 software (Waters, Milliford, MA, USA). Phosphoric acid solution at 50% (v/v) in acetonitrile was used for instrument calibration.

### DLS Assays

DLS measurements were performed using a Zetasizer Nano S DLS device from Malvern Instruments (Malvern, Worcestershire, UK) with a 633 nm laser, backscatter angle of 173° mode, thermostated with a Peltier system, and using a plastic micro cuvette. Aβ_1–42_ at 50 μM in 50 mM NaCl and 20 mM phosphate buffer, pH 7.4, at 25 °C alone or at 1:1 metal complex molar ratios was kept under stirring. Size distributions by intensity were determined in automatic mode at regular time intervals over a period of 10 min for each measurement. Thirteen acquisitions were recorded, each of 10 s in duration.

### MTT Assays

MCF7 and SH-SY5Y cells were grown at 37 °C in a humidified atmosphere of 5% CO_2_ in Eagle’s minimum essential medium (EMEM, Sigma-Aldrich MCF7) and Dulbecco’s Modified Eagle Medium and Ham’s:F12 (DMEM/F-12, SH-SY5Y) containing 10% fetal bovine serum (FBS), 100 μg mL^–1^l-glutamine, and 100 U mL^–1^ penicillin/streptomycin. MCF7 and SH-SY5Y cells were seeded in duplicates in 96-well plates at a density of 8000 cells/well and 25000 cells/well, respectively, and allowed to adhere overnight. Aβ peptides (stock solutions: 200 μM Aβ_1–42_ and 400 μM Aβ_21–40_ in 50 mM phosphate buffer at pH 7.4) in the absence and in the presence of the metal complexes at a 1:1 peptide to metal complex molar ratio (after 0, 2, and 24 h of stirring) were diluted in cell culture medium at a final concentration of 25 μM for Aβ_1–42_ and 50 μM Aβ_21–40_ and added to the cells for 24 h. Control cells were incubated with phosphate buffer diluted in cell culture medium at the same final concentration used for the Aβ peptides. After the incubation, MTT assays were used according to the manufacturer’s instructions. Briefly, 200 μL of the MTT labeling reagent (final concentration 0.5 mg/mL; Sigma-Aldrich) was added to each well for 3 h. Then, the supernatant was removed and substituted with 200 μL of isopropanol for 10 min at 37 °C, 5% CO_2_. The optical density of each well sample was determined at 570 nm by using a microplate reader. A blank absorbance value of 0.056, obtained from wells without cells but treated with MTT reagent, was subtracted from all the absorbance values. Then, the average absorbance value of cells treated with amyloid beta (Aβ) peptides was normalized to those of control cells incubated with the buffer, and cell viability was expressed as a percentage of the control. Standard deviations of normalized absorbance values were calculated via error propagation.

### SEM Microscopy

SEM micrographs were obtained using an Ultra Plus FESEM scanning electron microscope (Zeiss, Germany). 50 μL of a peptide solution at 100 μM alone and with compound **1** at two different ratios (1:1 and 1:5) in 50 mM phosphate buffer was mounted on microscope stubs, dried overnight, and sputter coated with a 7 nm thickness of gold.^79,80^

## Conclusions

Diruthenium compounds with paddlewheel structures and metal–metal multiple bonds have been extensively studied for medicinal purposes. They have shown unique electronic and magnetic properties and potential applications in catalysis and cancer therapy. In this study, we analyze, for the first time, the ability of two diruthenium complexes (**1** and **2**) to modulate the aggregation of Aβ peptides, key in Alzheimer’s disease. These complexes differ in their charges and steric hindrances around the Ru_2_ core. These differences are crucial for their mechanism of action in the suppression of amyloid aggregation. Only compound **1** favors the interaction between the bimetallic unit and the polypeptide chain. This is probably due to the different charges of the species that compounds **1** and **2** form in solution. Indeed, compound **2** forms anionic complexes in solution that make the approximation of the peptide backbone more difficult. However, as previously observed in its interaction with the model protein HEWL,^46^ some carbonate ligands can be substituted over time, and thus, it could coordinate similar residues of the chain, especially Asp. Our experimental data suggest that compound **1** can coordinate His side chains, probably at the axial site. The axial coordination accelerates the substitution of ligands at the equatorial positions that can be replaced by carboxylates of Asp/Glu residues. The π–π interactions arising from the formamidinate ligand of compound **1** and the aromatic residues of the peptide chain could also play a key role in the stabilization of the complex/peptide adduct. This dual and synergistic mechanism of action explains the greater effect exhibited by compound **1** as an inhibitor of Aβ-aggregation, which is more evident in experiments involving the entire polypeptide Aβ_1–42_. Both a “polydentate” chelating effect and π–π interactions exerted by the longest Aβ_1–42_ toward compound **1** can explain the DLS results. Indeed, only compound **1** determines a deep rearrangement of the polypeptide chain of Aβ_1–42_ leading to oligomeric states quite different from that of Aβ_1–42_ alone to the point of completely suppressing its cellular toxicity. With these results, we want to highlight the novel application of bimetallic Ru-based drugs as inhibitors of Aβ aggregation with important future impacts in drug discovery associated with Alzheimer’s disease.

## Supporting Information Available

Figure S1: Time course of ThT fluorescence emission intensity of compounds **1** and **2**; Figure S2: CD spectra of compounds **1** and **2**; Figure S3: ^1^H NMR spectra of Aβ_21–40_ in the absence and in the presence of compound **1** or compound **2**; Figure S4: Absorption spectra of compounds **1** and **2** upon the addition of increasing amount of Aβ peptides; Figure S5: Fluorescence emission spectra of Aβ_1–16_ in the absence and presence of compound **1** and compounds **1** and **2** alone; Figure S6: ESI-MS spectrum of Aβ_21–40_ alone or incubated with compound **1**; Figure S7: ESI-MS spectrum of Aβ_1–42_ alone or incubated with compound **1**; Figure S8: ESI-MS spectrum of Aβ_1–16_ alone or incubated with compound **2**; Figure S9: ESI-MS spectrum of Aβ_21–40_ alone or incubated with compound **2**; Figure S10: ESI-MS spectrum of Aβ_1–42_ alone or incubated with compound **2**; Table S1: Experimental *m*/*z* values detected in the spectra of Aβ peptides alone and with the addition of compound **1**; Table S2: Experimental *m*/*z* values detected in the spectra of Aβ peptides alone and with the addition of compound **2**; Table S3: SEM analysis of Aβ peptides in the presence and in the absence of compound **1** (PDF)
