# Supplementary material for: A Diruthenium Metallodrug as a Potent Inhibitor of Amyloid-β Aggregation: Synergism of Mechanisms of Action
Source: Inorg Chem. 2023 Dec 20;63(1):564–75. doi: 10.1021/acs.inorgchem.3c03441 (PMC10777406; doi:10.1021/acs.inorgchem.3c03441)
Supplement: Supplementary file 1 — ic3c03441_si_001.pdf [file ic3c03441_si_001.pdf]

SUPPORTING INFORMATION

A diruthenium metallodrug as a potent inhibitor of Amyloid-β aggregation: synergism of mechanisms of action

Sara La Manna[1], Concetta Di Natale[2], Valeria Panzetta[2,3], Marilisa Leone[4], Flavia A. Mercurio[4], Irene Cipollone[5,6], Maria Monti[5,6], Paolo A. Netti[2,3], Giarita Ferraro[5], Aarón Terán [7], Ana E. Sánchez-Peláez[7], Santiago Herrero[7], Antonello Merlino[5], Daniela Marasco [1,4,]*

[1]Department of Pharmacy, University of Naples "Federico II", 80131, Naples, Italy

[2]Department of Chemical, Materials, and Industrial Production Engineering (DICMaPI), University of Naples Federico II, 80125 Naples, Italy.

[3]Interdisciplinary Research Centre on Biomaterials (CRIB), University of Naples Federico II, Istituto Italiano di Tecnologia, 80125, Naples, Italy

[4]Institute of Biostructures and Bioimaging - CNR, 80145, Naples, Italy

[5]Department of Chemical Sciences, University of Naples "Federico II", 80126, Naples, Italy

[6]CEINGE Biotecnologie Avanzate "Franco Salvatore" S.c.a r.l., 80131, Naples, Italy

[7]MatMoPol Research Group, Department of Inorganic Chemistry, Faculty of Chemical Science, Complutense University of Madrid, Avenida Complutense s/n, 28040, Madrid, Spain.

Index

Figure S1. Time course of ThT fluorescence emission intensity of compound **1** and **2**. Values are the average of two measurements. **4**

Figure S2. CD spectra of compound 1 (panel A) and compound 2 (panel B), at the indicated times of stirring. **5**

Figure S3. ^{1}H NMR spectra of Aβ_{21-40} in the absence (blue) and in the presence of compound **1** (green) or compound **2** (red). H_N/aromatic and Hα/side chains regions of the spectra are shown on the left and on the right panels, respectively. **6**

Figure S4. Absorption spectra of compound **1** (left panel) and **2** (right panel) upon the addition of increasing amount of (a and b) Aβ_{1-42}, (c and d) Aβ_{1-16}, and (e and f) Aβ_{21-40} peptides. The arrows indicate the spectral variations. As insets, UV intensities at indicated wavelengths versus concentration of Aβ peptides are reported. **7**

Figure S5. (a-c) Fluorescence emission spectra at different times of Aβ_{1-16} in the absence and presence of compound **1**. (d-e) Fluorescence emission spectra at different times of compounds **1** and **2** (λ_{ex} = 440 nm)........ **8**

Figure S6. ESI-MS spectrum of Aβ_{21-40} peptide at 0 h (panel A). ESI-MS spectra of Aβ_{21-40} peptide incubated with compound **1** after 0 (panel B) and 24 h (panel C). The peaks marked with b_n derive from spontaneous in source fragmentation of Aβ_{21-40} peptide (b series elements); the asterisk highlighted the species present in the control (compound **1** alone). **9**

Figure S7. ESI-MS spectrum of Aβ_{1-42} peptide at 0 h (panel A). ESI-MS spectra of Aβ_{1-42} peptide incubated with compound **1** after 0 (panel B) and 24 h (panel C). The peaks marked with b_n derive from spontaneous in source fragmentation of Aβ_{1-42} peptide (b series elements); the asterisk highlighted the species present in the control (compound **1** alone). **10**

Figure S8. ESI-MS spectrum of Aβ_{1-16} peptide alone at 0 h (panel A). ESI-MS spectra of Aβ_{1-16} peptide incubated with compound **2** at 0 h (panel B) and at 24 h (panel C) conditions. The peaks marked with b_n derive from spontaneous in source fragmentation of Aβ_{1-16} peptide (b series elements). § indicates K^+ adducts. **1*1***

Figure S9. ESI-MS spectrum of Aβ_{21-40} peptide alone at 0 h (panel A). ESI-MS spectra of Aβ_{21-40} peptide incubated with compound **2** at 0 h (panel B) and at 24 h (panel C) conditions. The peaks marked with b_n derive from spontaneous in source fragmentation of Aβ_{21-40} peptide (b series elements). The § indicates K^+ adducts **1*2***

Figure S10. ESI-MS spectrum of Aβ_{1-42} peptide alone at 0 h (panel A). ESI-MS spectra of Aβ_{1-42} peptide incubated with compound **2** at 0 h (panel B) and at 24 h (panel C) conditions. The peaks marked with b_n derive from spontaneous in source fragmentation of Aβ_{1-42} peptide (b series elements); the asterisk highlighted the species present in the control (compound **2** alone). The § indicates K^+ adducts. **..13**

Table S1: Experimental m/z values detected in the spectra of Aβ_{1-16}, Aβ_{21-40}, Aβ_{1-42} alone (Control) and with the addition of compound **1** at 0 and 24 h of incubation. The ion species corresponding to each experimental m/z, the expected m/z value (theoretical) and their charge states are also reported. **..14**

Table S2: Experimental m/z values detected in the spectra of Aβ_{1-16}, Aβ_{21-40}, Aβ_{1-42} alone (Control) and with the addition of compound **2** at 0 and 24h of incubation. The ion species corresponding to each experimental m/z, the expected m/z value (theoretical) and their charge states are also reported. **..15**

Table S3: SEM analysis. Average diameter and length of fibers obtained for Aβ peptides in the presence and in the absence of compound **1**. **..16**

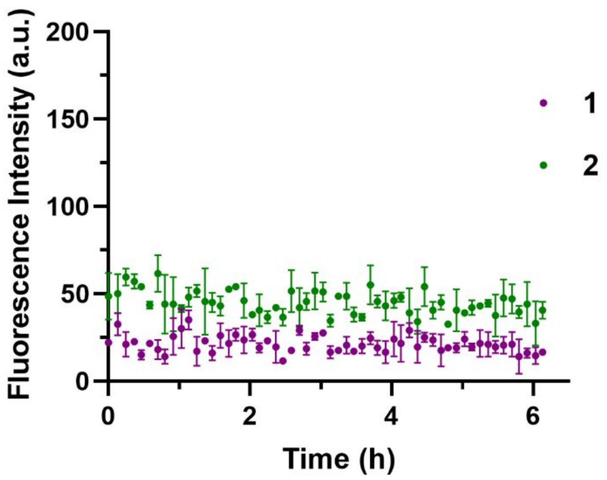

Figure S1. Time course of ThT fluorescence emission intensity of compound **1** and **2**. Values are the average of two measurements.

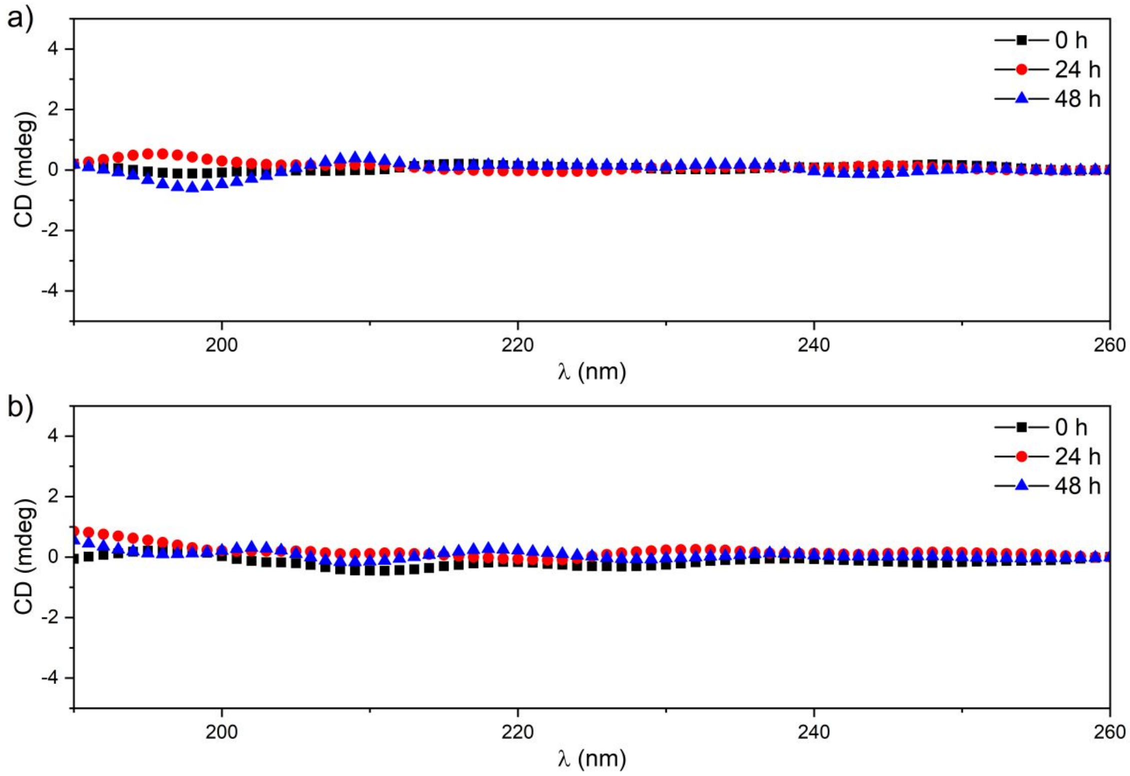

Figure S2. CD spectra of compound **1** (panel A) and compound **2** (panel B), at the indicated times of stirring.

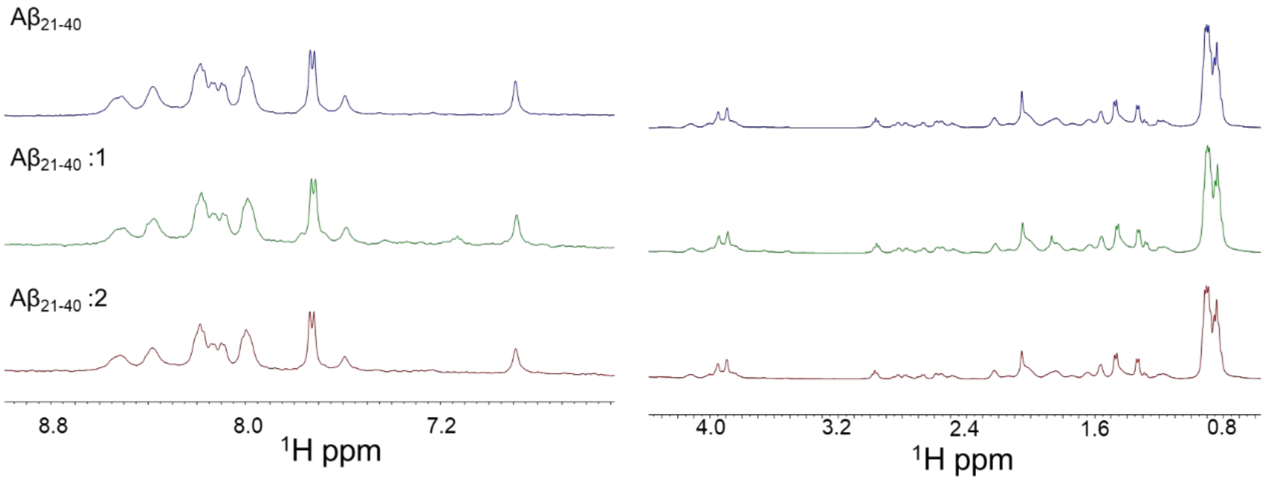

Figure S3. ^{1}H NMR spectra of A$\beta_{21\text{-}40}$ in the absence (blue) and in the presence of compound **1** (green) or compound **2** (red). H_N/aromatic and Hα/side chains regions of the spectra are shown on the left and on the right panels, respectively.

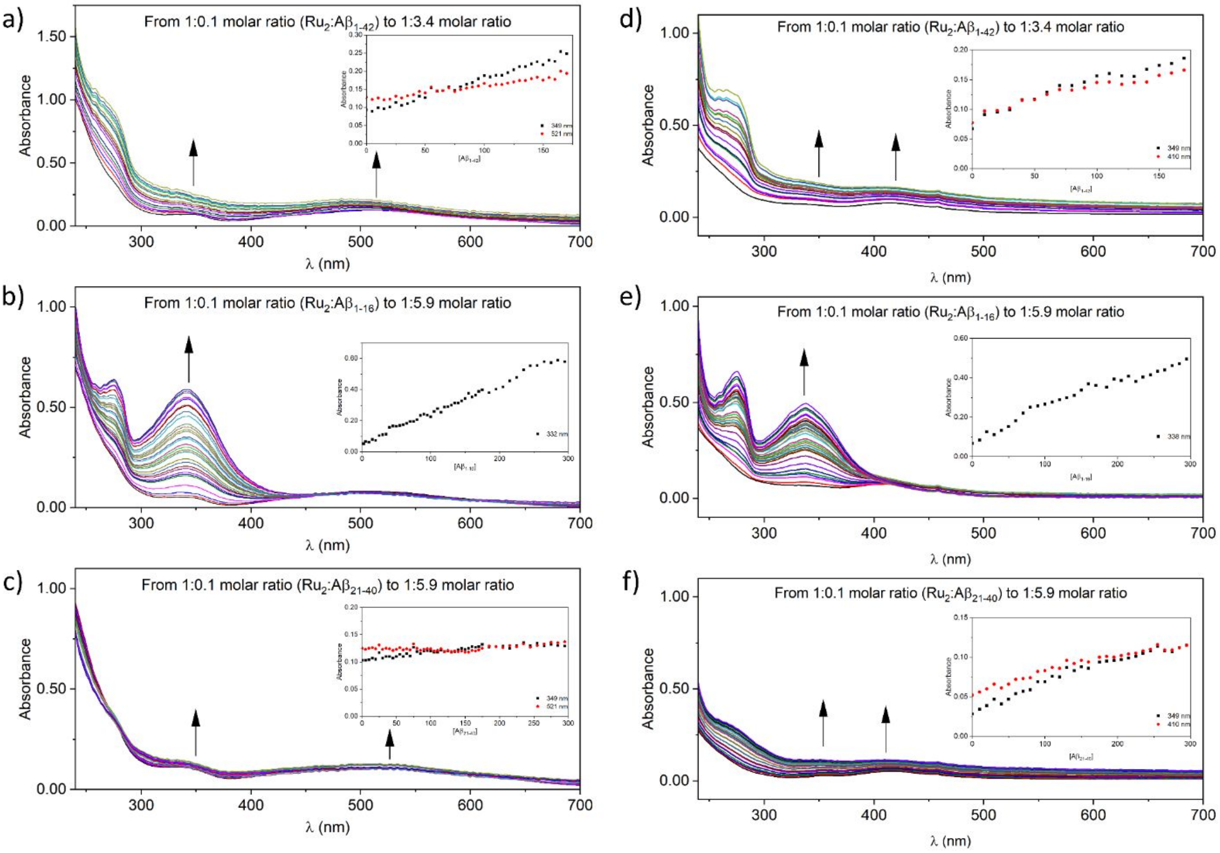

Figure S4. Absorption spectra of compound **1 (**left panel) and **2** (right panel) upon the addition of increasing amount of (a and b) $A\beta_{1--42}$, (c and d) $A\beta_{1--16}$, and (e and f) $A\beta_{21--40}$ peptides. The arrows indicate the spectral variations. As insets, UV intensities at indicated wavelengths versus concentration of Aβ peptides are reported.

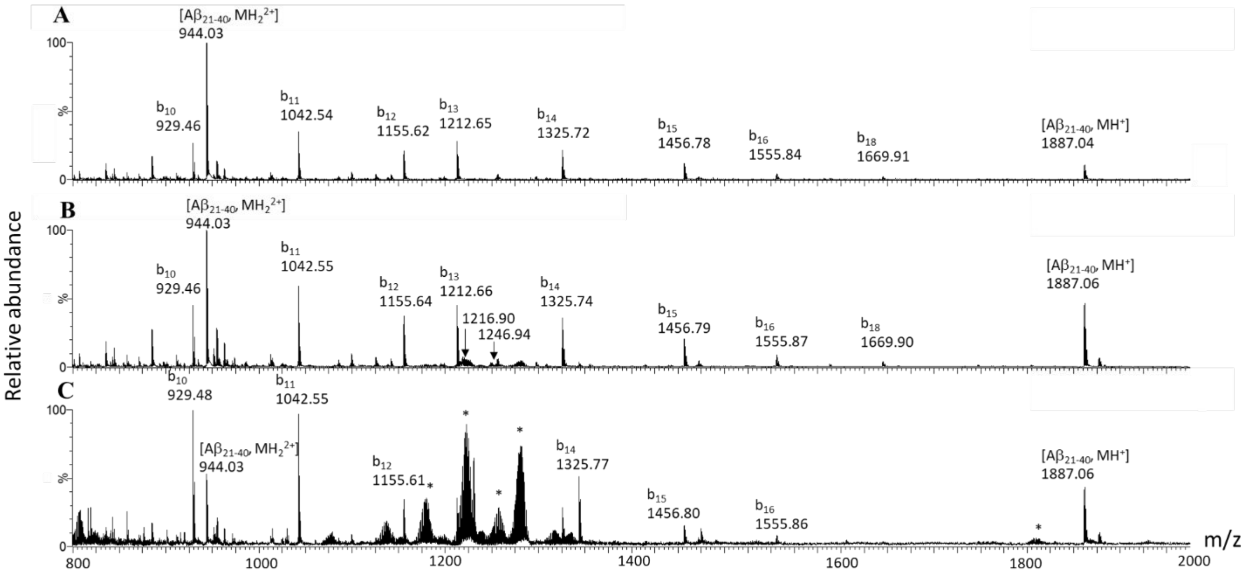

Figure S5. ESI-MS spectrum of A$\beta_{21\text{-}40}$ peptide at 0 h (panel A). ESI-MS spectra of A$\beta_{21\text{-}40}$ peptide incubated with compound **1** after 0 (panel B) and 24 h (panel C). The peaks marked with b_n derive from spontaneous in source fragmentation of A$\beta_{21\text{-}40}$ peptide (b series elements); the asterisk highlighted the species present in the control (compound **1** alone).

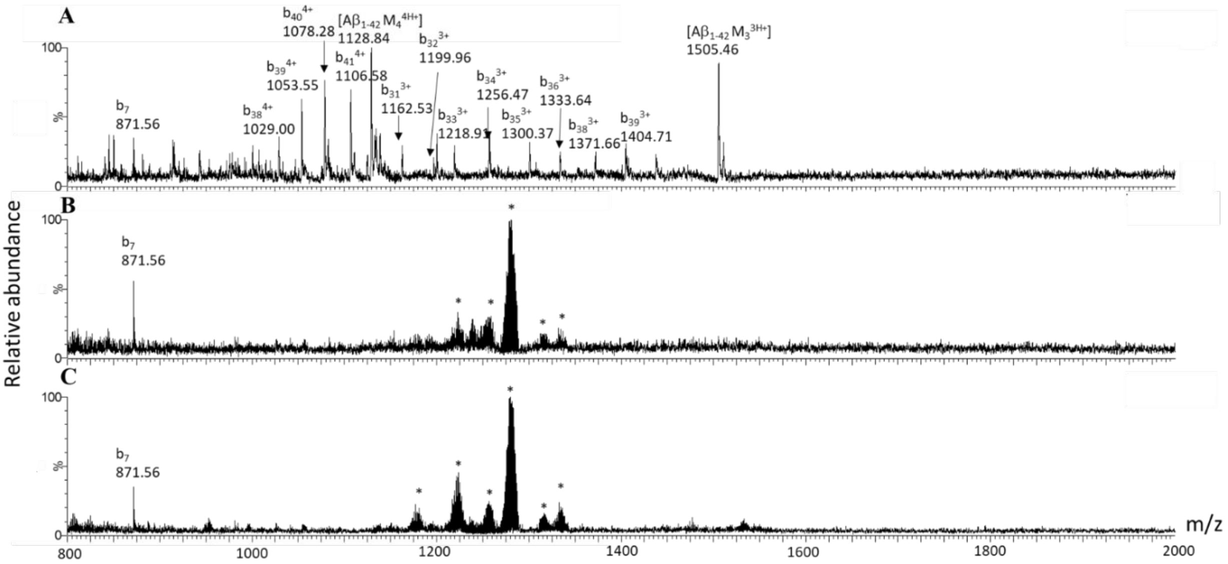

Figure S6. ESI-MS spectrum of A$\beta_{1\text{-}42}$ peptide at 0 h (panel A). ESI-MS spectra of A$\beta_{1\text{-}42}$ peptide incubated with compound **1** after 0 (panel B) and 24 h (panel C). The peaks marked with b_n derive from spontaneous in source fragmentation of A$\beta_{1\text{-}42}$ peptide (b series elements); the asterisk highlighted the species present in the control (compound **1** alone).

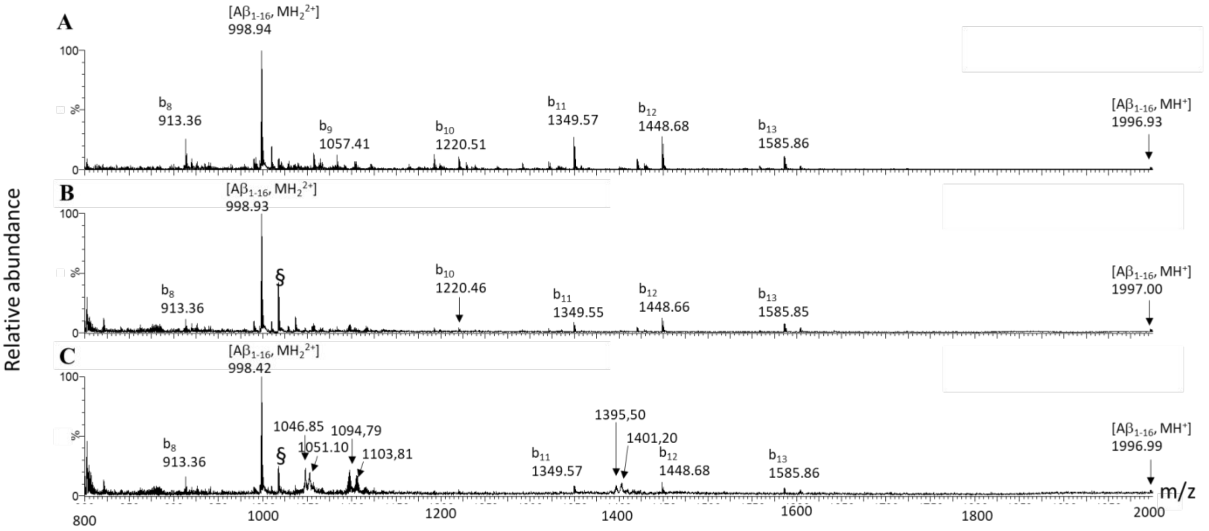

Figure S7. ESI-MS spectrum of Aβ_{1-16} peptide alone at 0 h (panel A). ESI-MS spectra of Aβ_{1-16} peptide incubated with compound **2** at 0 h (panel B) and at 24 h (panel C) conditions. The peaks marked with b_n derive from spontaneous in source fragmentation of Aβ_{1-16} peptide (b series elements). § indicates K^+ adducts.

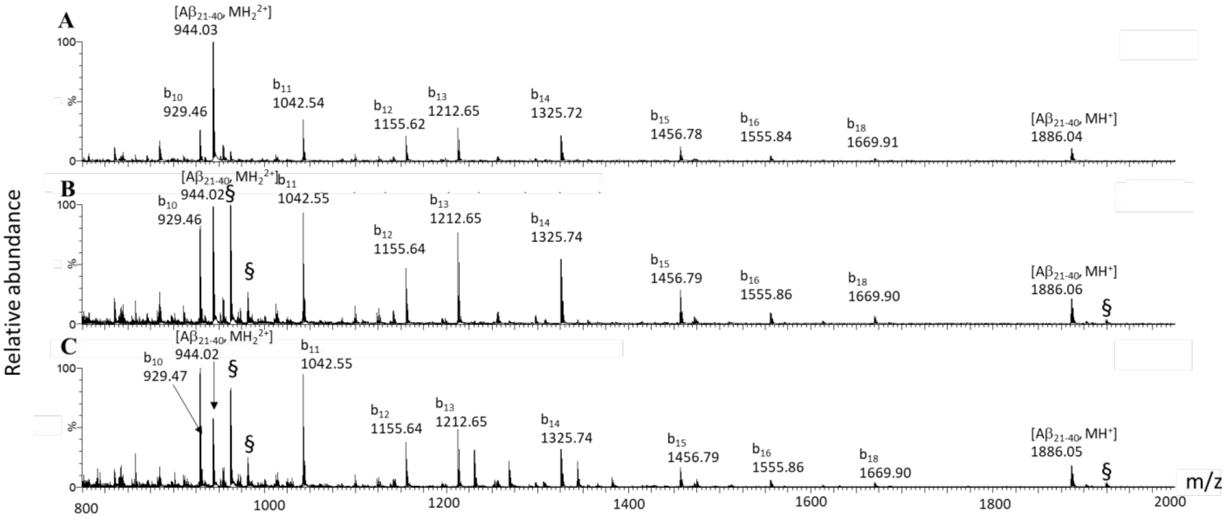

Figure S8. ESI-MS spectrum of $A\beta_{21-40}$ peptide alone at 0 h (panel A). ESI-MS spectra of $A\beta_{21-40}$ peptide incubated with compound **2** at 0 h (panel B) and at 24 h (panel C) conditions. The peaks marked with b_n derive from spontaneous in source fragmentation of $A\beta_{21-40}$ peptide (b series elements). The § indicates K^+ adducts.

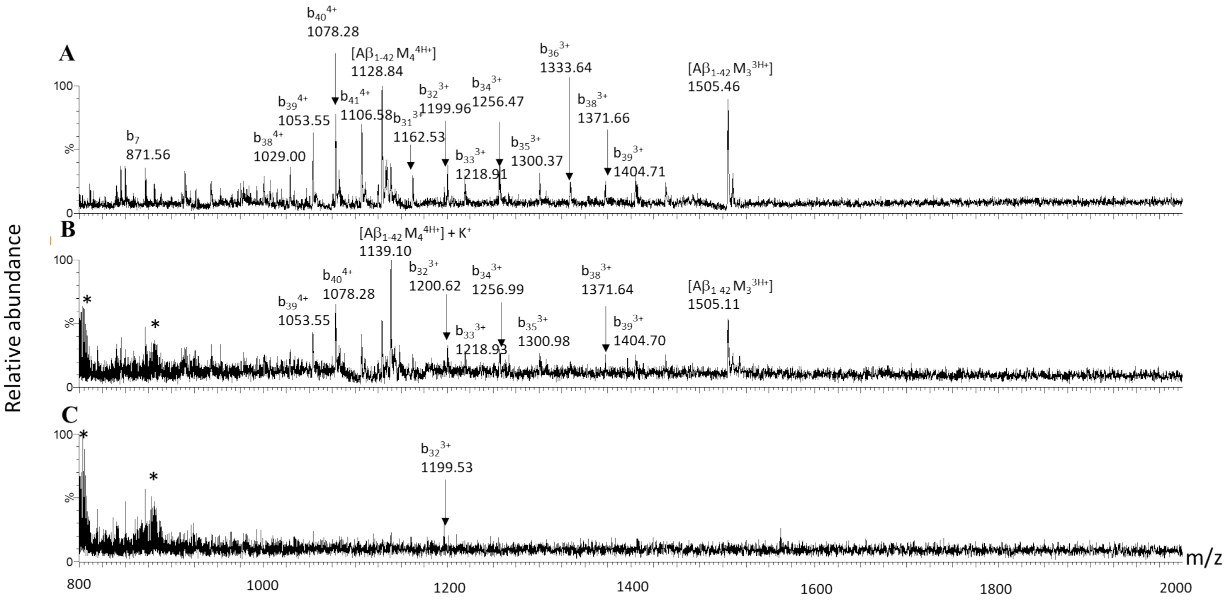

Figure S9. ESI-MS spectrum of A$\beta_{1\text{-}42}$ peptide alone at 0 h (panel A). ESI-MS spectra of A$\beta_{1\text{-}42}$ peptide incubated with compound **2** at 0 h (panel B) and at 24 h (panel C) conditions. The peaks marked with b_n derive from spontaneous in source fragmentation of A$\beta_{1\text{-}42}$ peptide (b series elements); the asterisk highlighted the species present in the control (compound **2** alone). The § indicates K^+ adducts.

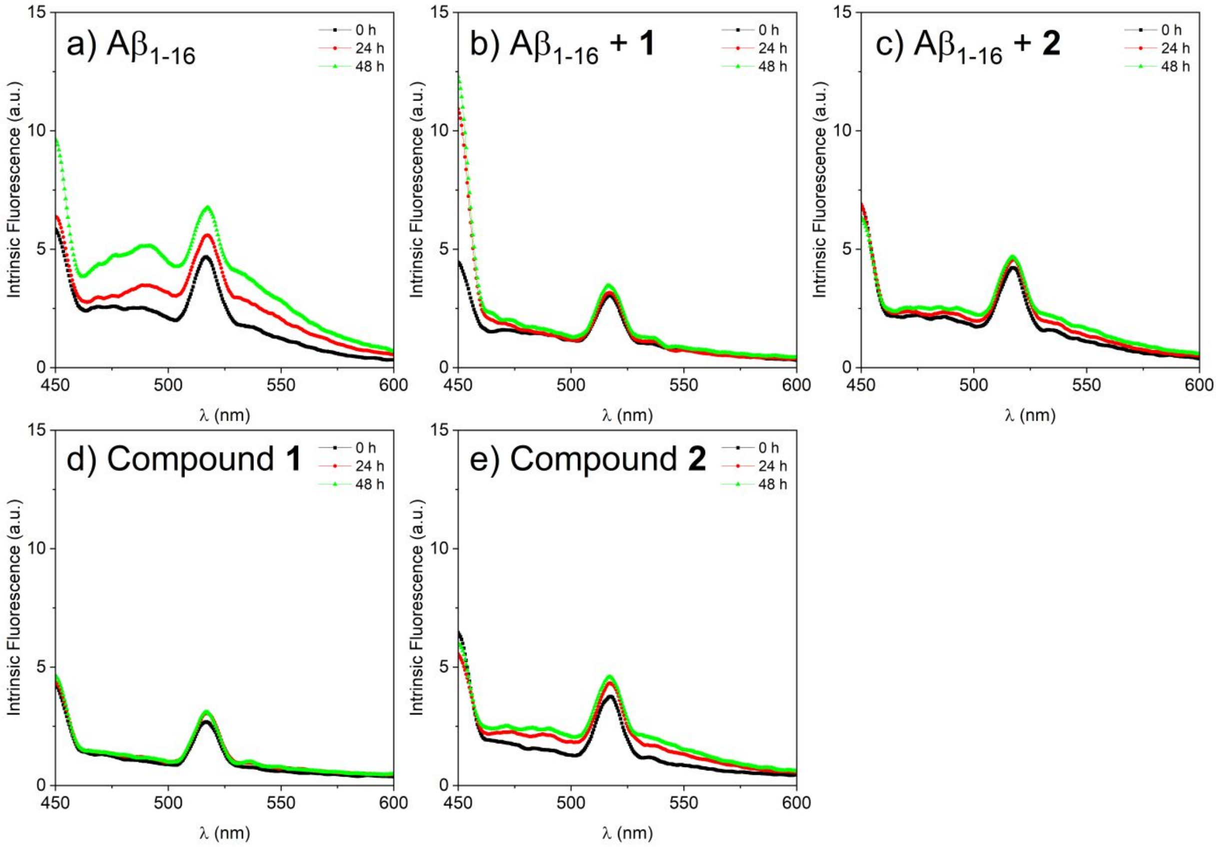

Figure S10. (a-c) Fluorescence emission spectra at different times of A$\beta_{1\text{-}16}$ in the absence and presence of compound **1**. (d-e) Fluorescence emission spectra at different times of compounds **1** and **2** (λ_{ex} = 440 nm).

Table S1: Experimental *m/z* values detected in the spectra of $A\beta_{1-16}$, $A\beta_{21-40}$, $A\beta_{1-42}$ alone (Control) and with the addition of compound **1** at 0 and 24 h of incubation. The ion species corresponding to each experimental *m/z*, the expected *m/z* value (theoretical) and their charge states are also reported.

	**Description**	***m/z* (charge)**			**Theoretical *m/z***
		**Control**	**0 h**	**24 h**	
$A\beta_{1-16}$:# 1	$A\beta_{1-16}$	1997.96 (+1)<br>998.94 (+2)	1997.98 (+1)<br>998.92 (+2)	-	1997.10<br>999.03
	b_{13}	1585.86 (+1)	-	-	1585.67
	b_{12}	1448.68 (+1)	1448.66 (+1)	-	1448.61
	b_{11}	1349.57 (+1)	-	-	1349.54
	b_{10}	1220.51 (+1)	-	-	1220.50
	b_9	1057.41 (+1)	-	-	1057.43
	b_8	913.36 (+1)	-	-	913.38
	$A\beta_{1-16}$ + **1** - Cl^- - H_2O - 2·CH_3COO^-	-	827.56 (+3)<br>1240.86 (+2)	827.56 (+3)<br>1240.88 (+2)	827.68<br>1241.01
	$A\beta_{1-16}$ + **1** - Cl^- - H_2O - CH_3COO^-	-	847.90 (+3)<br>1270.87 (+2)	847.94 (+3)<br>1270.89 (+2)	847.34<br>1270.52
	$A\beta_{1-16}$ + 2·(**1** - Cl^- - H_2O - 2·CH_3COO^-)	-	1485.87 (+2)	1485.90 (+2)	1485.00
	$A\beta_{1-16}$ + 2·(**1** - Cl^- - H_2O - 3/2·CH_3COO^-)	-	1514.99 (+2)	1515.03 (+2)	1514.50
	$A\beta_{1-16}$ + 2·(**1** - Cl^- - H_2O - CH_3COO^-)	-	1545.97 (+2)	1545.98 (+2)	1545.00
	$A\beta_{1-16}$ + 2·(**1** - Cl^- - H_2O - 1/2·CH_3COO^-)	-	1573.90 (+2)	1573.96 (+2)	1573.50
$A\beta_{21-40}$: #1	$A\beta_{21-40}$	1887.04 (+1)<br>944.03 (+2)	1887.06 (+1)<br>944.03 (+2)	1887.06 (+1)<br>944.03 (+2)	1887.22<br>944.11
	b_{18}	1669.91 (+1)	1669.90 (+1)	-	1669.86
	b_{16}	1555.84 (+1)	1555.87 (+1)	1555.86 (+1)	1555.81
	b_{15}	1456.78 (+1)	1456.79 (+1)	1456.80 (+1)	1456.75
	b_{14}	1325.72 (+1)	1325.74 (+1)	1325.77 (+1)	1325.71
	b_{13}	1212.65 (+1)	1212.66 (+1)	-	1212.62
	b_{12}	1155.62 (+1)	1155.64 (+1)	1155.61 (+1)	1155.60
	b_{11}	1042.54 (+1)	1042.55 (+1)	1042.55 (+1)	1042.52
	b_{10}	929.46 (+1)	929.46 (+1)	929.48 (+1)	929.43
	$A\beta_{21-40}$ + **1** - Cl^- - H_2O - CH_3COO^-	-	1216.90 (+2)	-	1216.08
	$A\beta_{21-40}$ + **1** - Cl^- - H_2O	-	1246.94 (+2)		1246.58
$A\beta_{1-42}$: #1	$A\beta_{1-42}$	1505.46 (+3)<br>1128.84 (+4)	-	-	1505.71<br>1129.54
	b_{41}	1106.58 (+4)			1106.56
	b_{40}	1078.28 (+4)			1078.29
	b_{39}	1404.71 (+3)<br>1053.55 (+4)	-	-	1404.37<br>1053.52
	b_{38}	1371.66 (+3)<br>1029.00 (+4)	-	-	1371.34<br>1028.78
	b_{36}	1333.64 (+3)	-	-	1333.33
	b_{35}	1300.37 (+3)	-	-	1300.31
	b_{34}	1256.47 (+3)	-	-	1256.63
	b_{33}	1218.91 (+3)	-	-	1218.92
	b_{32}	1199.96 (+3)	-	-	1199.92
	b_{31}	1162.53 (+3)	-	-	1162.23
	b_7	871.56 (+1)	871.56 (+1)	871.56 (+1)	871.56

Table S2: Experimental *m/z* values detected in the spectra of Aβ$_{1-16}$, Aβ$_{21-40}$, Aβ$_{1-42}$ alone (Control) and with the addition of compound **2** at 0 and 24h of incubation. The ion species corresponding to each experimental *m/z*, the expected *m/z* value (theoretical) and their charge states are also reported.

	**Description**	***m/z* (charge)**			**Theoretical *m/z***
		**Control**	**0 h**	**24 h**	
Aβ$_{1-16}$# 2	Aβ$_{1-16}$	1996.93 (+1)<br>998.94 (+2)	1997.00 (+1)<br>998.93 (+2)	1996.99 (+1)<br>998.92 (+2)	1997.10<br>999.03
	b$_{13}$	1585.86 (+1)	1585.85 (+1)	1585.86 (+1)	1585.67
	b$_{12}$	1448.68 (+1)	1448.66 (+1)	1448.68 (+1)	1448.61
	b$_{11}$	1349.57 (+1)	1349.55 (+1)	1349.57 (+1)	1349.54
	b$_{10}$	1220.51 (+1)	1220.46 (+1)	-	1220.50
	b$_{9}$	1057.41 (+1)	-	-	1057.43
	b$_{8}$	913.36 (+1)	913.36 (+1)	913.36 (+1)	913.38
	Aβ$_{1-16}$ + # 2 - ??	-	-	1094.79 (+2)	??
	Aβ$_{1-16}$ + #2 - ??	-	-	1103.81 (+2)	??
	2***Aβ$_{1-16}$*** + # 2 - ??$^{-}$	-	-	1046.85 (+4) 1395.50 (+3)	??<br>??
	2Aβ$_{1-16}$ + # 2 - ??	-	-	1051.10 (+4)<br>1401.20 (+3)	??<br>??
Aβ$_{21-40}$ # 2	Aβ$_{21-40}$	1887.04 (+1)<br>944.03 (+2)	1887.06 (+1)<br>944.02 (+2)	1887.05 (+1)<br>944.02 (+2)	1887.22<br>944.11
	b$_{18}$	1669.91 (+1)	1669.90 (+1)	1669.90 (+1)	1669.86
	b$_{16}$	1555.84 (+1)	1555.86 (+1)	1555.86 (+1)	1555.81
	b$_{15}$	1456.78 (+1)	1456.79 (+1)	1456.79 (+1)	1456.75
	b$_{14}$	1325.72 (+1)	1325.74 (+1)	1325.74 (+1)	1325.71
	b$_{13}$	1212.65 (+1)	1212.65 (+1)	1212.65 (+1)	1212.62
	b$_{12}$	1155.62 (+1)	1155.64 (+1)	1155.64 (+1)	1155.60
	b$_{11}$	1042.54 (+1)	1042.55 (+1)	1042.55 (+1)	1042.52
	b$_{10}$	929.46 (+1)	929.46 (+1)	929.47 (+1)	929.43
Aβ$_{1-42}$# 2	Aβ$_{1-42}$	1505.46 (+3)<br>1128.84 (+4)	1505.11 (+3)<br>1128.84 (+4)	-	1505.71<br>1129.54
	b$_{41}$	1106.58 (+4)	1106.58 (+4)		1106.56
	b$_{40}$	1078.28 (+4)	1078.28 (+4)		1078.29
	b$_{39}$	1404.71 (+3)<br>1053.55 (+4)	1404.70 (+3)<br>1053.55 (+4)	-	1404.37<br>1053.52
	b$_{38}$	1371.66 (+3)<br>1029.00 (+4)	1371.64 (+3)<br>-	-	1371.34<br>1028.78
	b$_{36}$	1333.64 (+3)	-	-	1333.33
	b$_{35}$	1300.37 (+3)	1300.98 (+3)	-	1300.31
	b$_{34}$	1256.47 (+3)	1256.99 (+3)	-	1256.63
	b$_{33}$	1218.91 (+3)	1218.93 (+3)	-	1218.92
	b$_{32}$	1199.96 (+3)	1200.62 (+3)	1199.53 (+3)	1199.92
	b$_{31}$	1162.53 (+3)	-	-	1162.23
	b$_{7}$	871.56 (+1)	-	-	871.37

Table S3: SEM analysis. Average diameter and length of fibers obtained for Aβ peptides in the presence and in the absence of compound **1.**

**Sample**	**Time**	**Average Length (µm)**	**Average Diameter (µm)**
$A\beta_{1\text{-}16}$ alone	*0*	*n.d.*	*n.d.*
	24	*n.d.*	*n.d.*
$A\beta_{1\text{-}16}$ + **1**	*0*	*n.d.*	*n.d.*
	24	*n.d.*	*n.d.*
$A\beta_{1\text{-}42}$ alone	0	444 ± 7	16 ± 4
	24	494 ± 11	12 ± 3
$A\beta_{1\text{-}42}$ + **1**	0	363 ± 9	13 ± 2
	24	*n.d.*	*n.d.*
$A\beta_{21\text{-}40}$ alone	0	273 ± 11	9 ± 2.
	24	359 ± 6	14 ± 4
$A\beta_{21\text{-}40}$ + **1**	0	363 ± 9	16 ± 4
	24	*n.d.*	*n.d.*
